# Sodium thiosulfate acts as a hydrogen sulfide mimetic to prevent intimal hyperplasia via inhibition of tubulin polymerisation

**DOI:** 10.1016/j.ebiom.2022.103954

**Published:** 2022-03-22

**Authors:** Diane Macabrey, Alban Longchamp, Michael R. MacArthur, Martine Lambelet, Severine Urfer, Sebastien Deglise, Florent Allagnat

**Affiliations:** a Department of Vascular Surgery, Lausanne University Hospital, Switzerland; b CHUV-Service de chirurgie vasculaire, Department of Biomedical Sciences, University of Lausanne, Bugnon 7A, Lausanne 1005, Switzerland; c Department of Health Sciences and Technology, Swiss Federal Institute of Technology (ETH) Zurich, Zurich, Switzerland

**Keywords:** Intimal hyperplasia, Smooth muscle cells, Proliferation, Hydrogen sulfide, Sodium thiosulfate, 3-MST/MPST, 3-mercaptopyruvate sulfurtransferase, ALAT, alanine amino-transferase, ASAT, aspartate amino-transferase, BSA, bovine serum albumin, CAS, carotid artery stenosis, *Cse/Cth*, cystathionine gamma lyase, CBS, cystathionine-β-synthase, CK, creatine kinase, CK-MB, creatine Kinase MB Isoenzyme, CUA, calcific uremic arteriopathy, DAPI, 4′,6-diamidino-2-phénylindole, DATS, dially trisulfide, DCB, drug-coated balloons, DES, drug-eluting stents, DMF, dimethyl formamide, ECM, extra-cellular matrix (), FBS, foetal bovine serum, H_2_S, hydrogen sulfide, IH, intimal hyperplasia, NaHS, sodium hydrogen sulfur, P4HA1, prolyl 4-hydroxylase alpha polypeptide I, PBS, phosphate buffered saline, PCNA, proliferating cell nuclear antigen, SBP, systolic blood pressure, STS, sodium thiosulfate, VSMC, vascular smooth muscle cells, VGEL, Van Gieson elastic lamina, TST, thiosulfate sulfurtransferase, SUOX, sulfite oxidase, LDLR, Low density lipoprotein receptor

## Abstract

**Background:**

Intimal hyperplasia (IH) remains a major limitation in the long-term success of any type of revascularisation. IH is due to vascular smooth muscle cell (VSMC) dedifferentiation, proliferation and migration. The gasotransmitter Hydrogen Sulfide (H_2_S), mainly produced in blood vessels by the enzyme cystathionine- γ-lyase (CSE), inhibits IH in pre-clinical models. However, there is currently no H_2_S donor available to treat patients. Here we used sodium thiosulfate (STS), a clinically-approved source of sulfur, to limit IH.

**Methods:**

Low density lipoprotein receptor deleted (LDLR^−/−^), WT or *Cse*-deleted (Cse^−/−^) male mice randomly treated with 4 g/L STS in the water bottle were submitted to focal carotid artery stenosis to induce IH. Human vein segments were maintained in culture for 7 days to induce IH. Further *in vitro* studies were conducted in primary human vascular smooth muscle cells (VSMCs).

**Findings:**

STS inhibited IH in WT mice, as well as in LDLR^−/−^ and Cse^−/−^ mice, and in human vein segments. STS inhibited cell proliferation in the carotid artery wall and in human vein segments. STS increased polysulfides *in vivo* and protein persulfidation *in vitro,* which correlated with microtubule depolymerisation, cell cycle arrest and reduced VSMC migration and proliferation.

**Interpretation:**

STS, a drug used for the treatment of cyanide poisoning and calciphylaxis, protects against IH in a mouse model of arterial restenosis and in human vein segments. STS acts as an H_2_S donor to limit VSMC migration and proliferation via microtubule depolymerisation.

**Funding:**

This work was supported by the Swiss National Science Foundation (grant FN-310030_176158 to FA and SD and PZ00P3-185927 to AL); the Novartis Foundation to FA; and the Union des Sociétés Suisses des Maladies Vasculaires to SD, and the Fondation pour la recherche en chirurgie vasculaire et thoracique.

**Research in context**

**Evidence before this study**

Intimal hyperplasia (IH) is a complex process leading to vessel restenosis, a major complication following cardiovascular surgeries and angioplasties. Therapies to limit IH are currently limited. Pre-clinical studies suggest that hydrogen sulfide (H_2_S), an endogenous gasotransmitter, limits restenosis. However, despite these potent cardiovascular benefits, H_2_S-based therapeutics are not available. Sodium thiosulfate (Na_2_S_2_O_3_) is a FDA-approved drug used for the treatment of cyanide poisoning and calciphylaxis, a rare condition of vascular calcification affecting patients with end-stage renal disease. Evidence suggest that thiosulfate may generate H_2_S *in vivo* in pre-clinical studies.

**Added value of this study**

Here, we demonstrate that STS inhibits IH in a surgical mouse model and in human vein *ex vivo*. We further found that STS increases circulating polysulfide levels *in vivo* and inhibits IH via disruption of the normal cell's cytoskeleton, leading to decreased cell proliferation and migration. Finally, STS rescues *Cse* knockout mice with impaired endogenous H_2_S production from accelerated IH formation.

**Implications of all the available evidence**

These findings suggest that STS holds translational potentials to limit IH following vascular surgeries and should be investigated in clinical trials.

Alt-text: Unlabelled box

## Introduction

Prevalence of peripheral arterial disease continues to rise worldwide, largely due to the combination of aging, smoking, hypertension, and diabetes mellitus.^1^, ^2^, ^3^ Open or endo-vascular surgery remains the best treatment when daily life activities are compromised despite exercise therapy or in threatened limb.^4^ However, the vascular trauma associated with any vascular surgery eventually lead to secondary occlusion of the injured vessel. Re-occlusive lesions result in costly and complex recurrent end-organ ischemia, and often lead to loss of limb, brain function, or life. Despite the advent of new medical devices such as drug eluting stent (DES) and drug-coated balloons (DCB), restenosis has been delayed rather than suppressed, and stents still suffer from high rates of in-stent restenosis.^5^ Plus, the use of DES and DCB prolongs the need for anti-thrombotic therapies, with their associated risk of haemorrhages. In December 2018, Katsanos and colleagues reported, in a systematic review and meta-analysis, an increased risk of all-cause mortality following application of paclitaxel‐coated balloons and stents in the femoropopliteal artery.^6^ These findings were recently confirmed by other groups using the same data.^7^^,^^8^ However, other meta-analyses did not find any association between paclitaxel devices and long-term survival, despite similar target populations and vessel segments.^9^, ^10^, ^11^, ^12^, ^13^ Overall, these reports question the widespread use of paclitaxel for the treatment of restenosis, and supports the development of other approaches or use of other molecules. While the focal nature of restenotic lesions prompts the use of local treatment, systemic approaches for the treatment of restenosis still remains an option.

Restenosis is mainly due to intimal hyperplasia (IH), a process instated by endothelial cell injury and inflammation, which induces vascular smooth muscle cell (VSMC) reprogramming. VSMCs become proliferative and migrating, secrete extra-cellular matrix (ECM) and form a new layer called the neo-intima, which slowly reduces the vessel luminal diameter.^14^

Hydrogen sulfide (H_2_S) is a gasotransmitter derived from cysteine metabolism, produced endogenously through the transsulfuration pathway by 3 main enzymes, cystathionine-γ-lyase (CSE), cystathionine-β-synthase (CBS) and 3-mercaptopyruvate sulfurtransferase (3-MST).^15^ Mice lacking *Cse* develop more IH in after carotid ligation, and are rescued by H_2_S supplementation.^16^ Circulating H_2_S levels are reduced in humans suffering from vascular occlusive disease^17^^,^^18^ and pre-clinical studies using water-soluble sulfide salts such as Na_2_S and NaHS have shown that H_2_S has cardiovascular protective properties,^15^ including reduction of IH *in vivo* in rats,^19^ rabbits,^20^ mice,^21^ and *ex-vivo* in human vein segments.^22^ However, the fast and uncontrolled release, narrow therapeutical range and high salt concentration of these compounds limit their potential. Due to these limitations, H_2_S-based therapy are currently not available.

Here, we focused on sodium thiosulfate (Na_2_S_2_O_3_), a FDA-approved drug used in gram-quantity doses for the treatment of cyanide poisoning^23^ and calciphylaxis, a rare condition of vascular calcification affecting patients with end-stage renal disease.^24^ Pharmaceutical-grade sodium thiosulfate (STS) is available and thiosulfate has been suggested to release H_2_S through non-enzymatic and enzymatic mechanisms.^25^^,^^26^

We tested whether STS inhibits IH in a surgical mouse model and in an *ex vivo* model of IH in human vein culture. NaHS, a validated H_2_S donor, was systematically compared to STS. We observed that STS was at least as potent as NaHS to inhibit IH in our two models. STS increased protein persulfidation and circulating polysulfide levels *in vivo*. STS inhibited apoptosis and matrix deposition associated with the development of IH, as well as VSMC proliferation and migration. We further observed that STS and NaHS induced microtubule depolymerisation in VSMCs, which may explain the anti-proliferative effect of STS in those cells.

## Methods

For details on materials and reagents please see the Supplementary Table S1 and 2.

### Mouse treatment

WT mice C57BL/6JRj (RRID:MGI:5752053) mice were purchased form Janvier Labs (Le Genest-Saint-Isle, France). LDL receptor knock out (LDLR^−/−^) mice (*Ldlr^tm1Her^*, JAX stock #002207^27^; MGI Cat# 3611043, RRID:MGI:3611043), kindly provided by Prof. Caroline Pot (Lausanne university Hospital, Switzerland), were bred and housed in our animal facility and genotyped as previously described.^27^ All mice were housed at standard housing conditions (22 °C, 12  h light/dark cycle), with ad libitum access to water and regular diet (SAFE®150 SP-25 vegetal diet, SAFE diets, Augy, France). LDLR^−/-^ mice were put on a cholesterol rich diet (Western 1635, 0.2% Cholesterol, 21% Butter, U8958 Version 35, SAFE® Complete Care Competence) for 3 weeks prior to surgery. 8 to 12 weeks old male WT or LDLR^−/−^ mice were randomly divided into control vs. sodium thiosulfate (STS) or NaHS. Sodium thiosulfate was given in mice via the water bottle at 4 g/L (0.5 g/Kg/day), changed 3 times a week. NaHS was given in mice via the water bottle at 0.5 g/L (125 mg/Kg/day), changed every day. Mice were euthanized after 7 or 28 days of treatment by cervical dislocation under isoflurane anaesthesia (inhalation 2.5% isoflurane under 2.5 L of O_2_) followed by PBS perfusion. Aortas, carotid arteries, livers and serum or plasma (via intracardiac blood collection with a 24G needle) were harvested.

### Cse^−/−^ mice

*Cth* knockout (Cse^−/−^) mice were generated from a novel floxed line generated by embryonic injection of ES cells containing a Cth allele with LoxP sites flanking exon 2 (Cth^tm1a(EUCOMM)Hmgu;^ RRID:IMSR_:Cmsu10294). Both ES cells and recipient embryos were on C57BL/6J background. Mice that were homozygous for the floxed allele were crossed with CMV-cre global cre-expressing mice (B6.C-Tg(CMV-cre)1Cgn/J), which have been backcrossed with C57BL/6J for 10 generations to create constitutive whole-body CSE^−/−^ animals on a C57BL/6J background. The line was subsequently maintained by breeding animals heterozygous for the *Cth* null allele. Mouse ear biopsies were taken and digested in DirectPCR lysis reagent with proteinase K. WT, heterozygous and knockout mice were identified by PCR using the forward primer 5’-AGC ATG CTG AGG AAT TTG TGC-3’ and reverse primer 5’-AGT CTG GGG TTG GAG GAA AAA-3’ to detect the WT allele and the forward primer 5’-TTC AAC ATC AGC CGC TAC AG-3’ to detect knock-out allele using the platinum *Taq* DNA Polymerase.

### Carotid artery stenosis (CAS) surgery

The carotid artery stenosis (CAS) was performed as previously published^28^ on 8 to 10 weeks old male WT or LDLR^−/−^ mice. Treatment was initiated 3 days before surgery and continued for 28 days post-surgery until organ collection. The day of the surgery, mice were anaesthetised with an intraperitoneal injection of Ketamine (80 mg/kg) and Xylazine (15 mg/kg). The left carotid artery was exposed and separated from the jugular vein and vague nerve. Then, a 7.0 PERMA silk (Johnson & Johnson AG, Ethicon, Switzerland) thread was looped and tightened around the carotid in presence of a 35-gauge needle. The needle was removed, thereby restoring blood flow, albeit leaving a significant stenosis. The stenosis triggers IH proximal to the site of injury, which was measured 28 days post-surgery.^28^ Buprenorphine (0.05 mg/kg) was provided subcutaneously as post-operative analgesic every 12 h for 24 h. Mice were euthanized under isoflurane anaesthesia (inhalation 2.5% isoflurane under 2.5 L of O_2_) by cervical dislocation and exsanguination, perfused with PBS followed by buffered formalin 4% through the left ventricle. Surgeries were performed in a random order, alternating mice from different cages to minimise potential confounders such as the order of treatments and measurements, or animal/cage location. All series of surgeries included all the groups to be compared to minimise batch effects. Surgeons were blind to the group during surgeries.

### Human tissue and VSMC culture

Static vein culture was performed as previously described.^22^^,^^29^ Briefly, the vein was cut in 5 mm segments randomly distributed between conditions. One segment (D0) was immediately preserved in formalin or flash frozen in liquid nitrogen and the others were maintained in culture for 7 days in RPMI-1640 Glutamax I supplemented with 10% FBS and 1% antibiotic solution (10,000 U/mL penicillin G, 10,000 U/mL streptomycin sulphate) in cell culture incubator at 37 °C, 5% CO_2_ and 21% O_2_.

Human VSMCs were also prepared from these human great saphenous vein segments as previously described.^22^^,^^29^ Vein explants were plated on the dry surface of a cell culture plate coated with 1% Gelatine type B (Sigma-Aldrich). Explants were maintained in RPMI, 10% FBS medium in a cell culture incubator at 37 °C, 5% CO_2_, 5% O_2_ environment. 8 different veins/patients were used in this study to generate VSMC.

### Carotid and human vein histomorphometry

After 7 days in culture, or immediately upon vein collection (D0), the vein segments were fixed in buffered formalin, embedded in paraffin, cut into 5 µm sections, and stained using Van Gieson Elastic Laminae (VGEL) as previously described.^22^^,^^30^ Three photographs per section were taken at 100x magnification and 8 measurements of the intima and media thicknesses were made, evenly distributed along the length of the vein wall.

Left ligated carotids were isolated and paraffin-embedded. Six-µm sections of the ligated carotid artery were cut from the ligature towards the aortic arch and stained with VGEL for morphometric analysis. Cross sections at every 300 µm and up to 2 mm from the ligature were analysed using the Olympus Stream Start 2.3 software (Olympus, Switzerland). For intimal and medial thickness, 72 (12 measurements/cross section on six cross sections) measurements were performed, as previously described.^22^

Two independent researchers blind to the experimental groups did the morphometric measurements, using the Olympus Stream Start 2.3 software (Olympus, Switzerland).^22^

### H_2_S and polysulfide measurement

Free H_2_S was measured in cells using the SF_7_-AM fluorescent probe^31^ (Sigma-Aldrich). The probe was dissolved in anhydrous DMF at 5 mM and used at 5 μM in serum-free RPMI medium with or without VSMCs. Free polysulfide was measured in cells using the SSP4 fluorescent probe. The probe was dissolved in DMF at 10 mM and diluted at 10 μM in serum-free RPMI medium with or without VSMCs. Fluorescence intensity (λ_ex_ = 495 nm; λ_em_ = 520 nm) was measured continuously in a Synergy Mx fluorescent plate reader (BioTek Instruments AG, Switzerland) at 37 °C before and after addition of various donors, as indicated.

Plasma polysulfides were measured using the SSP4 fluorescent probe. Plasma samples were diluted 3 times and incubated for 10 min at 37 °C in presence of 10 μM SSP4. Plasma polysulfides were calculated using a Na_2_S_3_ standard curve. Liver polysulfides were measured using the SSP4 fluorescent probe. Pulverized frozen liver was resuspended in PBS-0.5% triton X-100, sonicated and adjusted to 0.5 mg/ml protein concentration. Lysates were incubated for 30 min at 37 °C in presence of 10 μM SSP4 and fluorescence intensity (λ_ex_ = 495 nm; λ_em_ = 520 nm) was measured in a Synergy Mx fluorescent plate reader (BioTek Instruments AG, Switzerland)

### Persulfidation protocol

Persulfidation protocol was performed using a dimedone-based probe as recently described.^32^ Persulfidation staining was performed on VSMCs grown on glass coverslips. Briefly, 1 mM 4-Chloro-7-nitrobenzofurazan (NBF-Cl, Sigma) was diluted in PBS and added to live cells for 20 min. Cells were washed with PBS then fixed for 10 min in ice-cold methanol. Coverslips were rehydrated in PBS, and incubated with 1 mM NBF-Cl for 1 h at 37 °C. Daz2-Cy5.5 (prepared with 1 mM Daz-2, 1 mM alkyne Cy5.5, 2 mM copper(II)-TBTA, 4 mM ascorbic acid with overnight incubation at RT, followed by quenching for 1h with 20 mM EDTA) was added to the coverslips and incubated at 37 °C for 1 h. After washing with methanol and PBS, coverslips were mounted in Vectashield mounting medium with DAPI and visualized with a 90i Nikon fluorescence microscope.

### BrdU assay

VSMCs were grown at 80% confluence (5·10^3^ cells per well) on glass coverslips in a 24-well plate and starved overnight in serum-free medium. Then, VSMC were either treated or not (ctrl) with the drug of choice for 24 h in full medium (RPMI 10% FBS) in presence of 10 µM BrdU. All conditions were tested in parallel. All cells were fixed in ice-cold methanol 100% after 24 h of incubation and immunostained for BrdU. Images were acquired using a Nikon Eclipse 90i microscope. BrdU-positive nuclei and total DAPI-positive nuclei were automatically detected using the ImageJ software.^22^

### Flow cytometry

VSMCs were grown at 70% confluence (5·10^4^ cells per well) and treated for 48 h with 15 mM STS or 10 nM Nocodazole. Then, cells were trypsinised, collected and washed in ice-cold PBS before fixation by dropwise addition of ice-cold 70% ethanol while slowly vortexing the cell pellet. Cells were fixed for 1 h at 4 °C, washed 3 times in ice-cold PBS and resuspended in PBS supplemented with 20 µg/mL RNAse A and 10 µg/mL DAPI. Flow cytometry was performed in a Cytoflex-S apparatus (Beckmann).

### Wound healing assay

VSMCs were grown at confluence (10^4^ cells per well) in a 12-well plate and starved overnight in serum-free medium. Then, a scratch wound was created using a sterile p200 pipette tip and medium was changed to full medium (RPMI 10% FBS). Repopulation of the wounded areas was recorded by phase-contrast microscopy over 24 h in a Nikon Ti2-E live cell microscope. All conditions were tested in parallel. The area of the denuded area was measured at *t* = 0 h and *t* = 10 h after the wound using the ImageJ software by two independent observers blind to the conditions. Data were calculated as a percentage of the wound closure.

### Immunohistochemistry

Polychrome Herovici staining was performed on paraffin sections as described.^33^ Young collagen was stained blue, while mature collagen was pink. Cytoplasm was counterstained yellow. Haematoxylin was used to counterstain nuclei blue to black.

Collagen III staining was performed on frozen sections (OCT embedded) of human vein segments using mouse anti-Collagen III antibody. Briefly, tissue slides were permeabilised in PBS supplemented with 2 wt. % BSA and 0.1 vol. % Triton X-100 for 30 min, blocked in PBS supplemented with 2 wt. % BSA and 0.1 vol. % Tween-20 for another 30 min, and incubated overnight with the primary antibody diluted in the same buffer. The slides were then washed 3 times for 5 min in PBS supplemented with 0.1 vol. % Tween-20, and incubated for 1 h at room temperature with anti-mouse AlexaFluor 568 (1/1000, ThermoFischer). Slides were visualised using a Nikon 90i fluorescence microscope (Nikon AG). Collagen III immunostaining area was quantified using the ImageJ software and normalised to the total area of the vein segment.

PCNA (proliferating cell nuclear antigen), P4HA, cleaved caspase 3 and α-tubulin immunohistochemistry was performed on paraffin sections.^34^ After rehydration and antigen retrieval (TRIS-EDTA buffer, pH 9, 17 min in a microwave at 500 W), immunostaining was performed on human vein or carotid sections using the EnVision®+ Dual Link System-HRP (DAB+) according to manufacturer's instructions. Slides were further counterstained with haematoxylin. PCNA and haematoxylin positive nuclei were manually counted by two independent observers blinded to the conditions.

α-tubulin immunofluorescent staining in human VSMCs was performed as previously described. Cell were fixed at -20 °C for 10 min in absolute methanol. Then, cells were blocked/permeabilised in PBS- triton 0.2%, BSA 3% for 45 min at room temperature. Cells were incubated overnight at 4 °C in the primary antibody diluted in PBS-0.1% tween, 3% BSA, washed 3 times in PBS and incubated for 1 h at room temperature with the secondary antibody diluted in PBS-0.1% tween, 3% BSA, washed again 3 times in PBS and mounted using Vectashield mounting medium for fluorescence with DAPI. The microtubule staining was quantified automatically using FiJi (ImageJ, 1.53c). Image processing was as follows: Plugin, Tubeness/Process, Make Binary/Analyze, Skeletonize 2D/3D. Data were summarised as filament number and total length, normalised to the number of cells per images. Data were generated from images from 3 independent experiments, 3 to 4 images per experiment per condition.

### Reverse transcription and quantitative polymerase chain reaction (RT-qPCR)

VSMCs were homogenised in Tripure Isolation Reagent (Roche, Switzerland), and total RNA was extracted according to the manufacturer's instructions. After RNA reverse transcription (Prime Script RT reagent, Takara), cDNA levels were measured by qPCR Fast SYBR™ Green Master Mix in a Quant Studio 5 Real-Time PCR System (Applied Biosystems, ThermoFischer Scientific), using the following primers: Homo sapiens thiosulfate sulfurtransferase (*TST*), forward:5’-GCTGGTGGATTCAAGGTCTCA-3’, Reverse: 5’-GACGGCACCACGGATATGG-3’; Homo sapiens sulfite oxidase (*SUOX*), forward: 5’-GGTGCAGTGTTGGCCTATCA-3’, Reverse: 5’-ACCCAGATCCCAGTCTCAGG-3’.

### Western blotting

Mice aortas or human vein segments were flash-frozen in liquid nitrogen, grinded to powder and resuspended in SDS lysis buffer (62.5 mM TRIS pH6,8, 5% SDS, 10 mM EDTA). Protein concentration was determined by DC protein assay. 10 to 20 µg of protein were loaded per well. Primary cells were washed once with ice-cold PBS and directly lysed with Laemmli buffer as previously described.^22^^,^^29^ Lysates were resolved by SDS-PAGE and transferred to a PVDF membrane Immobilon-P. Immunoblot analyses were performed as previously described^29^ using the antibodies described in supplementary Table S1. Membranes were revealed using Immobilon Western Chemiluminescent HRP Substate in an Azure Biosystems 280, and analysed using Image J. Protein abundance was normalised to total protein using Pierce™ Reversible Protein Stain Kit for PVDF Membranes.

### *In vitro* tubulin polymerization assay

The assay was performed using the *In Vitro* Tubulin Polymerization Assay Kit (≥99% Pure Bovine Tubulin), according to the manufacturer's instruction.

### Statistical analyses

All experiments adhered to the ARRIVE guidelines and followed strict randomisation. All experiments and data analysis were conducted in a blind manner using coded tags rather than actual group name. A power analysis was performed prior to the study to estimate sample-size. We hypothesized that STS or NaHS would reduce IH by 50%. Using an SD at +/- 20% for the surgery and considering a power at 0.9, we calculated that *n* = 12 animals/group was necessary to validate a significant effect of the STS or NaHS. Animals with pre-existing conditions (malocclusion, injury, abnormal weight) were not operated or excluded from the experiments upon discovery during dissection (kidney disease). A few animals died during surgery or did not recover from surgery and had to be euthanized before the end of the experiment. All experiments were analysed using GraphPad Prism 9. Normal distribution of the data was assessed using Shapiro-Wilk test and Kolmogorov-Smirnov test. All data had a normal distribution. One or 2-ways ANOVA were performed followed by multiple comparisons using post-hoc t-tests with the appropriate correction for multiple comparisons.

### Ethics statement

Human great saphenous veins were obtained from donors who underwent lower limb bypass surgery.^35^ Written, informed consent was obtained from all vein donors for human vein and VSMC primary cultures. The study protocols for organ collection and use were reviewed and approved by the Centre Hospitalier Universitaire Vaudois (CHUV) and the Cantonal Human Research Ethics Committee (http://www.cer-vd.ch/, no IRB number, Protocol Number 170/02), and are in accordance with the principles outlined in the Declaration of Helsinki of 1975, as revised in 1983 for the use of human tissues.

All animal experimentations were conformed to *the National Research Council:* Guide for the Care and Use of Laboratory Animals.^36^ All animal care, surgery, and euthanasia procedures were approved by the CHUV and the Cantonal Veterinary Office (SCAV-EXPANIM, authorisation number 3114, 3258 and 3504).

### Role of funding source

The funding sources had no involvement in study design, data collection, data analyses, interpretation, or writing of report.

## Results

### STS limits IH development in mice after carotid artery stenosis

**Figure 1:**
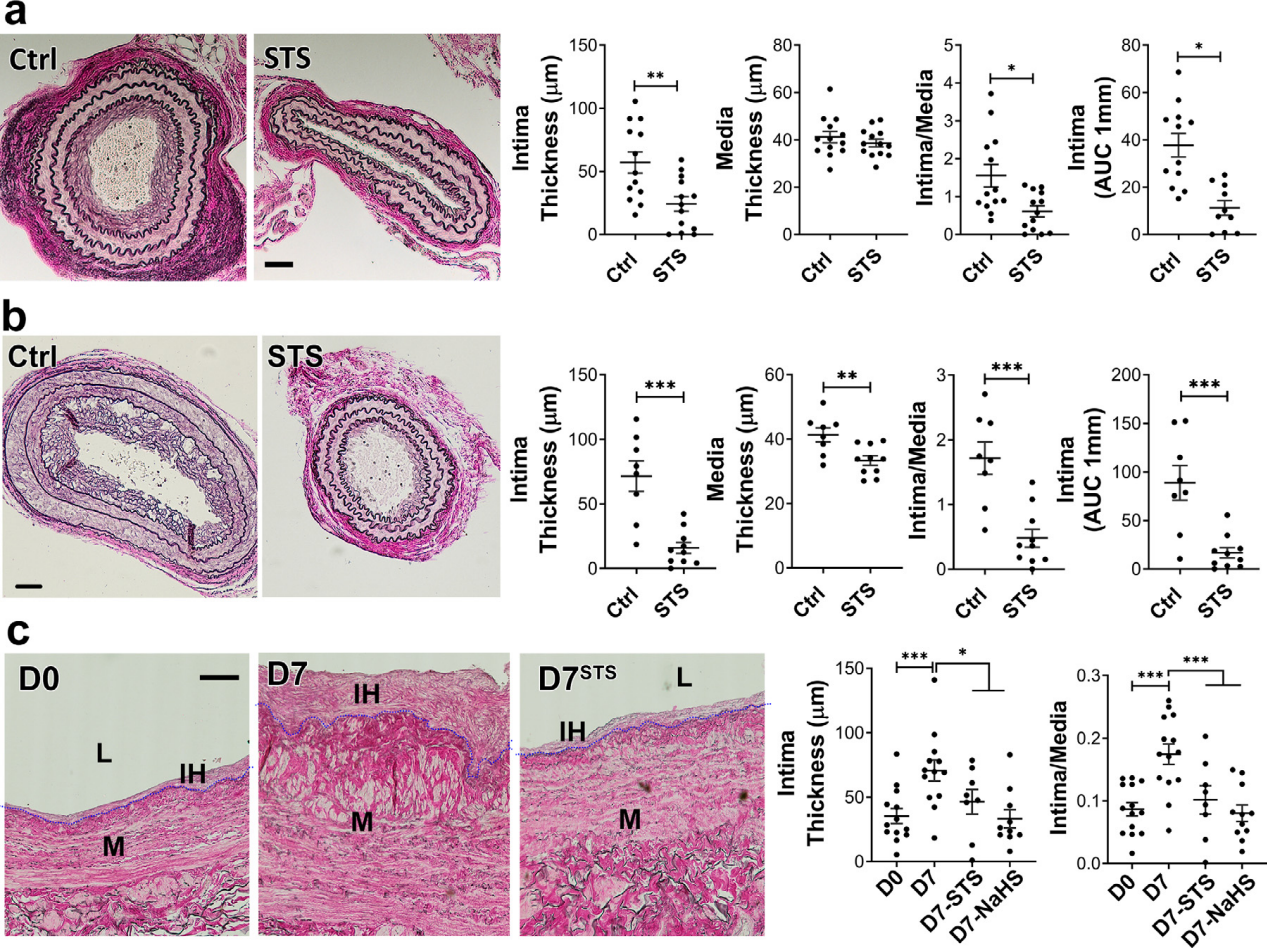
**STS decrease IH formation after carotid artery stenosis in mice and in cultured human saphenous veins.** a, b) WT (a) or LDLR^−/−^ mice (b) treated or not (ctrl) with 4 g/L STS were subjected to the carotid artery stenosis surgery. VGEL staining of left carotid cross sections and morphometric measurements of intima thickness, media thickness, intima over media ratio and intima thickness AUC. Scale bar 40 µm. Data are mean±SEM of 13 (c) and 8 (b) animals per group. *p < .05, **p < .01, ***p < .001 as determined by bilateral unpaired t-test. c) Intima thickness, media thickness and intima over media ratio of freshly isolated human vein segments (D0) or after 7 days (D7) in static culture with STS (15 mM) or NaHS (100 µm). Scale bar 60 µm. Data are mean ± SEM of 12 different veins/patients. *p < .05, **p < .01, ***p < .001, as determined by repeated measures one-way ANOVA with Dunnett's multiple comparisons.

We first assessed whether STS protects against IH as measured 28 days after mouse carotid artery stenosis (CAS).^28^ STS treatment (4 g/L) decreased IH by about 50% in WT mice (Figure 1a), as expressed as the mean intima thickness, the ratio of intima over media thickness (I/M), or the area under the curve of intima thickness calculated over 1 mm. We further tested the safety of systemic STS treatment by looking at several blood parameters, revealing that STS had no effect on kidney (urea), liver (ASAT, ALAT) and heart (CK, CK-MB) function. STS also had no effect on blood pH and HCO_3_ levels and blood chemistry (Na, Ca, K) levels (supplemental Table S3). To model the hyperlipidemic state of patients with PAD, we also performed the CAS model on hypercholesterolemic LDLR^−/−^ mice fed for 3 weeks with a cholesterol-rich diet. As expected, the LDLR^−/−^ mice developed more IH than WT mice upon CAS, and STS treatment lowered IH by about 70% (Figure 1b). Interestingly, STS did not reduce media thickness in WT mice (*p* = 0.39) but significantly reduced media thickness in LDLR^−/−^ mice (*p =* 0.0067; Figure 1b). STS had no effect on media thickness in native carotids of WT and LDLR^−/−^ mice (Figure S1 in the online supplementary files). Of note, the sodium salt H_2_S donor NaHS (0.5 g/L) also significantly decreased IH following carotid stenosis in WT mice (Figure S2).

### STS limits IH development in a model of *ex vivo* human vein segment culture

Both STS (15 mM) and NaHS (100 µM) inhibited IH development in our validated model of static *ex vivo* human vein segment culture^22^ as measured by intima thickness and I/M ratio (Figure 1c). The polysulfide/H_2_S donors diallyl trisulfide (DATS), cysteine-activated H_2_S donor 5a and GYY4137 also prevented the development of IH in human vein segments (Figure S3).

### STS is a biologically active source of sulfur

**Figure 2:**
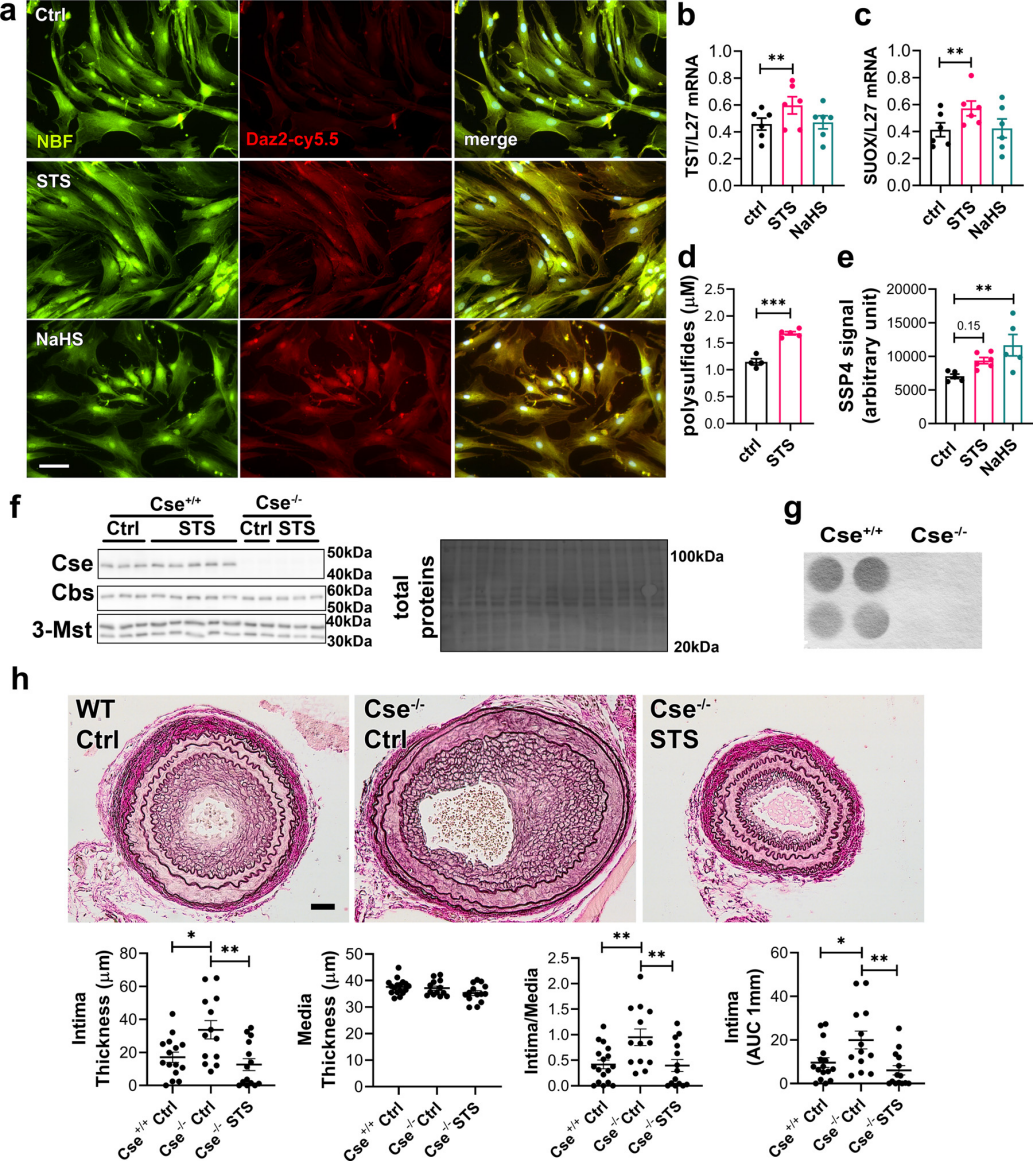
**STS increases protein persulfidation.** (a) *In situ* labelling of intracellular protein persulfidation assessed by DAz-2:Cy5.5 (red), normalized to NBF-adducts fluorescence (green), in VSMCs exposed for 4 h to NaHS (100 µm) or STS (15 mM). Representative images of 5 independent experiments. Scale bar 20 μM. b, c) Human *TST* and *SUOX* mRNA levels in VSMCs treated or not (Ctrl) for 24 h with NaHS (100 µm) or STS (15 mM). (d) Plasma polysulfides levels, as measured by the SSP4 probe, in mice treated 7 days with STS 4 g/L. Data are scatter plots with mean ± SEM of 4 animals per group. ***p < .001 as determined by bilateral unpaired t-test. (e) Polysulfides levels, as measured by the SSP4 probe in liver extracts of mice treated 7 days with STS 4 g/L or NaHS 0.5 g/L. Data are scatter plots with mean ± SEM of 5 animals per group. **p < .01 as determined by One-way ANOVA with post-hoc t-tests with Tukey's correction for multiple comparisons. (f) Western blot analysis of Cse, Cbs and 3-Mst in Cse^−/−^ and Cse^+/+^ mice, treated or not with STS 4 g/L for 4 weeks (3 to 5 animals per group). (g) Led acetate assay to measure Cse-mediated H_2_S production in Cse^−/−^ and Cse^+/+^ mice. Data are representative of 4 animals per group. (h) Intima thickness, media thickness, intima over media ratio and intima thickness AUC of CAS operated mice measured 28 days after surgery in Cse^+/+^ versus Cse^−/−^ mice treated or not (Cse^−/−^ Ctrl) with 4 g/L STS (Cse^−/−^ STS). Scale bar 50 µm. Data are scatter plots with mean ± SEM of 8 to 10 animals per group. *p < .05, **p < .01, as determined by one-way ANOVA with post-hoc t-tests with Tukey's correction for multiple comparisons.

Overall, STS and “classical” H_2_S donors similarly inhibit IH. To measure whether STS releases detectable amounts of H_2_S or polysulfides, we used the SF_7_-AM^31^ and SSP4 probes,^37^ respectively. We could not detect any increase in SF_7_-AM or SSP4 fluorescence in presence of STS with or without VSMCs. Na_2_S_3_ was used as a positive control for the SSP4 probe and NaHS as a positive control for the SF_7_-AM probe (Figure S4). The biological activity of H_2_S is mediated by post-translational modifications of reactive cysteine residues by persulfidation, which influence protein activity.^32^^,^^38^ As a proxy for H_2_S release, we assessed global protein persulfidation by DAZ-2-Cy5.5 labelling of persulfide residues in VSMCs treated for 4 h with NaHS or STS. Both STS and NaHS similarly increased persulfidation in VSMCs (Figure 2a). STS, but not NaHS, also increased the mRNA expression of *TST* and *SUOX* (Figure 2b, c), which are key enzymes of the H_2_S biosynthetic pathway and sulfide oxidizing unit involved in thiosulfate metabolism.^39^^,^^40^ Using the SSP4 probe, we further observed higher polysulfides levels *in vivo* in the plasma of mice treated for 1 week with 4 g/L STS (Figure 2d). Similarly, we observed a non-significant increase in polysulfides in the liver of mice treated with STS (*p =* 0.15). As a positive control, mice treated with 0.5 g/L NaHS had significantly higher polysulfides levels in the liver (Figure 2e). CSE is the main enzyme responsible for endogenous H_2_S production in the vasculature and Cse^−/−^ mice have been shown to develop more IH.^16^^,^^21^^,^^41^ To study the impact of STS on restenosis independently of endogenous H_2_S production, we generated a new Cse^−/−^ mouse line. As expected, these mice did not express *Cse* (Figure 2f), nor produced H_2_S in the liver (Figure 2g), which is the main CSE-expressing tissue. These mice did not display any growth defect (Figure S5a), had normal systolic blood pressure (Figure S5b) and carotid and aortic arteries were similar to Cse^+/+^ littermates (Figure S5c). Consistent with a role as an H_2_S donor, STS fully rescued Cse^−/−^ mice from increased IH in the model of carotid artery stenosis (Figure 2h).

### STS limits IH-associated matrix deposition and apoptosis in human vein segments

**Figure 3:**
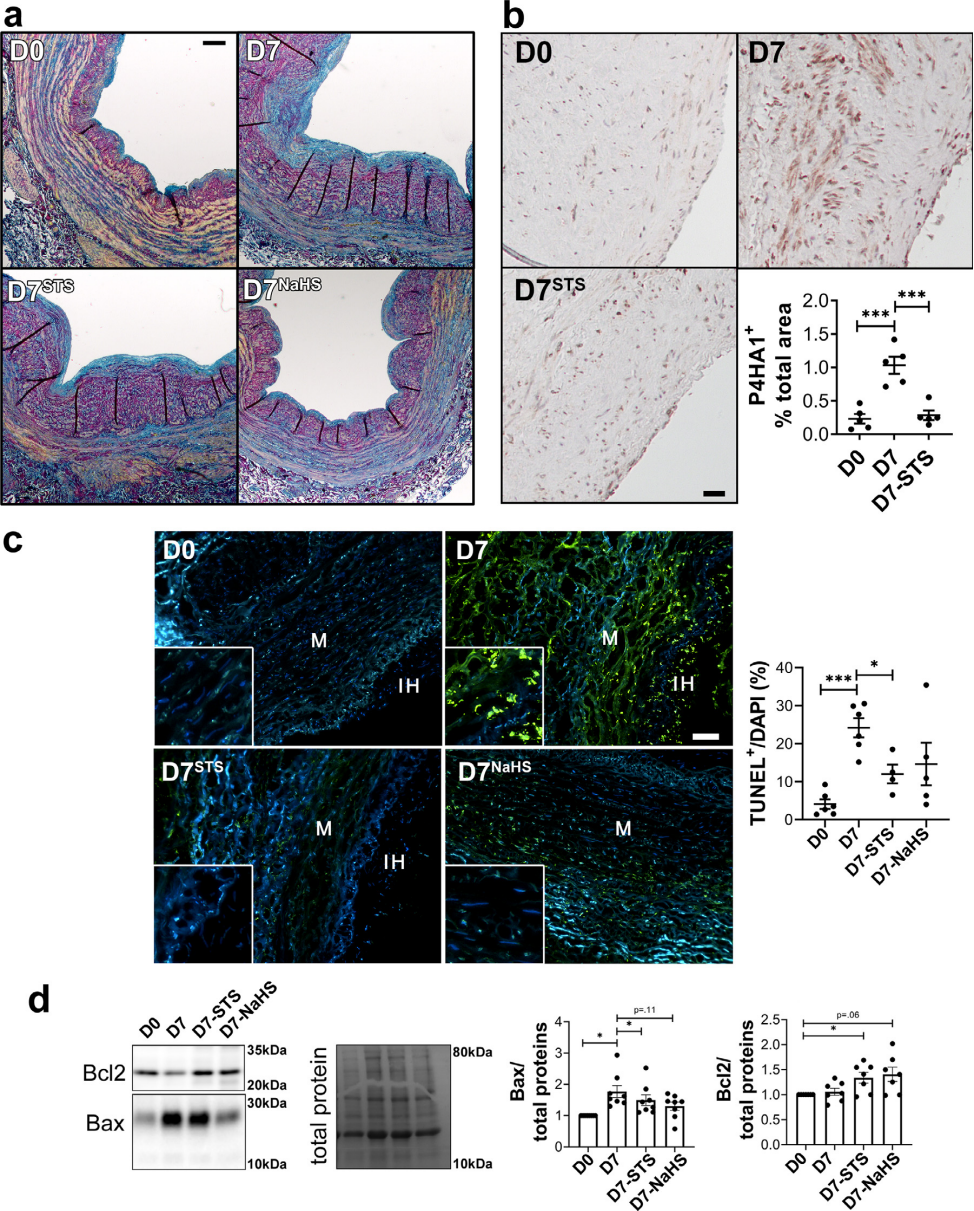
**STS decreases apoptosis and matrix deposition in human vein segments.** Human vein segment at day 0 (D0) or after 7 days of static culture with or without (D7) 15 mM STS or 100 µm NaHS. (a) Representative Herovici staining of 5 different human vein segments. Mature collagen I is stained pink; new collagen III is stained blue; cytoplasm is counterstained yellow; nuclei are stained blue to black. Scale bar=80 µm (b) *Left panels:* Representative P4HA1 staining. Scale bar=50 µm. *Right panel:* Quantitative assessment of P4HA1 staining. Data are scatter plots of 5 different veins with mean ± SEM. ***p < .01 as determined by paired repeated measures one-way ANOVA with Dunnett's multiple comparisons. (c) *Left panels:* Representative TUNEL staining in human vein segments. *Right panel:* Apoptosis is expressed as TUNEL positive (green) over DAPI positive nuclei. Scale bar= 50 µm. Data are scatter plots of 5 to 6 different veins with mean ± SEM. *p < .05, ***p < .001 as determined by mixed model analysis with Dunnett's multiple comparisons. (d) Representative western blot of Bax and Bcl2 over total protein and quantitative assessment of 7 different human veins. Data are scatter plots with mean ± SEM. *p < .05 as determined by repeated measures one-way ANOVA with Dunnett's multiple comparisons (For interpretation of the references to color in this figure legend, the reader is referred to the web version of this article.).

We further assessed matrix deposition and apoptosis in human vein segments. Concomitant with IH formation, *ex vivo* vein culture (D7) resulted in *de novo* collagen deposition compared to D0, as assessed by polychrome Herovici staining (Figure 3a). Analysis of immature collagen III levels confirmed that vein culture (D7) resulted in collagen deposition compared to D0, as assessed by immunostaining (Figure S6a) and Western blotting (Figure S6b). Furthermore, vein culture (D7) resulted in overexpression of the prolyl 4-hydroxylase alpha polypeptide I (P4HA1), which catalyses folding of collagen polypeptide chains into stable triple-helical molecules.^42^ Overall, STS and NaHS treatments reduced collagen deposition as assessed by Herovici staining, Collagen III and P4HA1 analyses (Figures 3 and S6). TUNEL assay revealed that STS, and to a lesser degree NaHS, decreased apoptosis observed after 7 days in culture (D7) (Figure 3c). STS also attenuated pro-apoptotic protein Bax overexpression observed after 7 days in culture, while NaHS decreased Bax level (*p =* .11; Figure 3d). STS and NaHS also increased the protein level of anti-apoptotic protein Bcl-2 (*p =* .06 and *p =* .04; Figure 3d). Of note, cleaved caspase 3 immunostaining in CAS-operated carotids in WT mice revealed that there is no detectable apoptosis in the carotid wall in this model 28 days after surgery (Figure S7).

### STS blocks VSMC proliferation and migration

**Figure 4:**
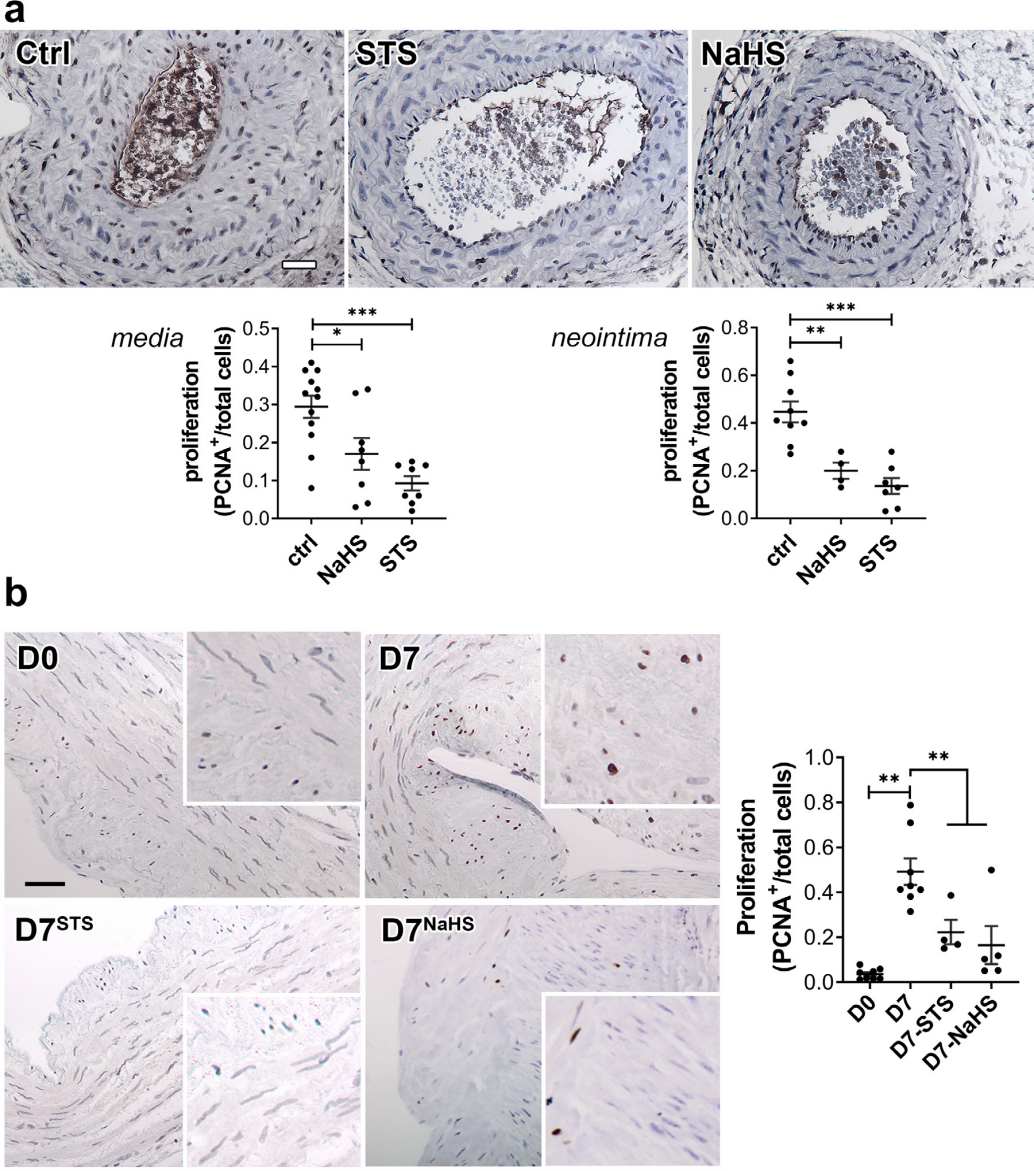
**STS inhibits cell proliferation*****in vivo*****in mouse carotids and*****ex-vivo*****in human vein segments.** PCNA immunostaining on CAS operated carotids in WT mice (a) treated or not (Ctrl) with STS 4 g/L or NaHS 0.5g/L for 28 days, and human vein segments (b) incubated or not (Ctrl) with 15 mM STS or 100 µm NaHS for 7 days. Proliferation is expressed as the ratio of PCNA positive (brown) nuclei over total number of nuclei. Data are scatter plots with mean±SEM. (a) Scale bar 20 µm. *p < .05, **p < .01, ***p < .001 as determined from 8 to 12 animals per group by one-way ANOVA with Dunnett's multiple comparisons. (b) Scale bar 50 µm **p < .01, as determined from 5 to 7 different veins by mixed model analysis and Dunnett's multiple comparisons.

IH is driven by VSMC reprogramming towards a proliferating, migrating and ECM-secreting phenotype.^14^ Both STS and NaHS significantly reduced the percentage of proliferating cells (defined as PCNA positive nuclei over total nuclei) *in vivo* in CAS-operated carotids in WT mice (Figure 4a) and *ex vivo* in human vein segments (Figure 4b).

**Figure 5:**
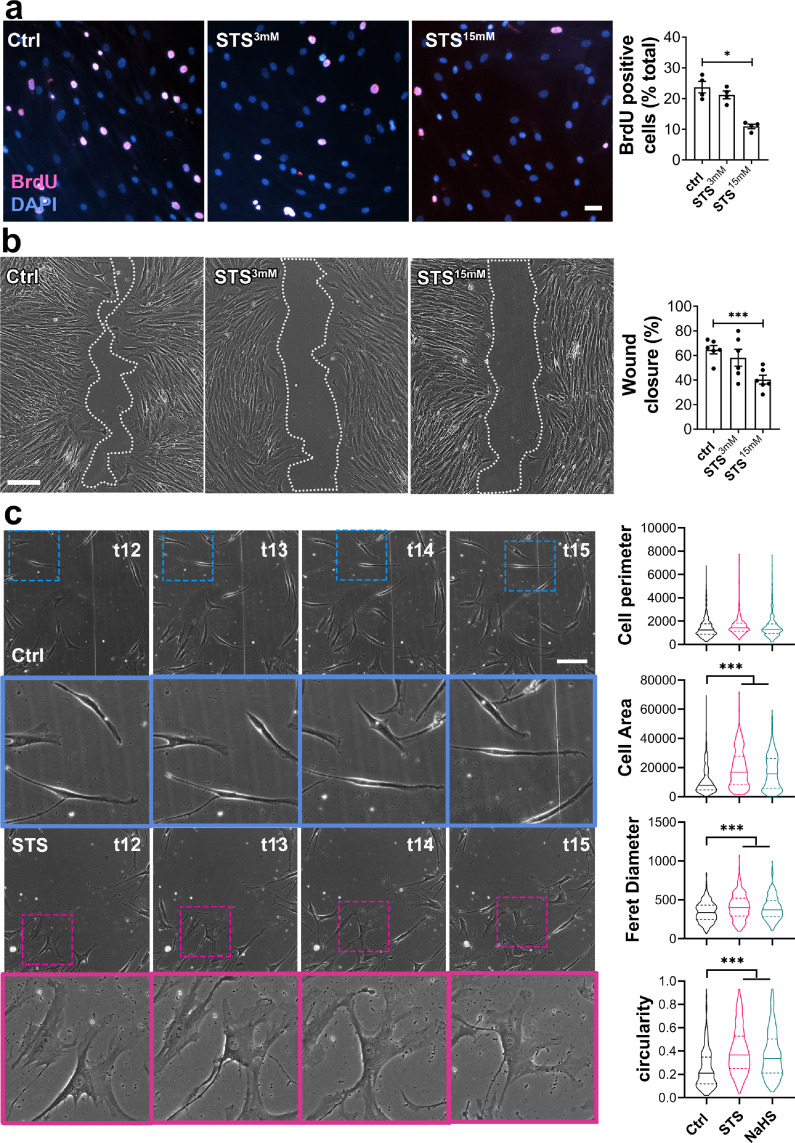
**STS inhibits VSMC proliferation and migration*****in vitro*****.** (a) VSMC proliferation (BrdU incorporation) in cells treated or not (Ctrl) for 24 h with 3 or 15 mM STS. Data are % of BrdU positive nuclei (pink) over DAPI positive nuclei. Scale bar: 25 µm. Data shown as mean ± SEM of 6 different experiments. *p < .05 as determined by repeated measures one-way ANOVA with Dunnett's multiple comparisons tests. (b) VSMC migration in cells treated or not (Ctrl) with 3 or 15 mM STS, as assessed by wound healing assay, expressed as the percentage of wound closure after 10 h. Scale bar: 100 µm. Data are scatter plots with mean ± SEM of 5 independent experiments in duplicates. ***p < .001 as determined by repeated measures one-way ANOVA with Dunnett's multiple comparisons. (c) Bright field images of VSMC morphology in cells exposed or not (Ctrl) to 15 mM STS or 100 µm NaHS, as measured as cell perimeter, cell area, Feret diameter and circularity index assessed during wound healing assay. Data are violin plots with median and quartiles (dotted lines) of 5 independent experiments. ***p < .001 as determined by one-way ANOVA with Dunnett's post-hoc test. Scale bar: 80 µm. Pink and blue insets are 3-fold magnifications of outlined areas.

*In vitro,* STS dose-dependently decreased primary human VSMC proliferation as assessed by BrdU incorporation assay (Figure 5a). Of Note, NaHS as well as DATS, H_2_S donor 5A and GYY4137, also decreased VSMC proliferation (Figure S8). STS and NaHS also decreased VSMC migration in a wound healing assay, in absence (Figure 5b), or in presence of the mitosis inhibitor mitomycin C (Figure S9). Further evaluation of cell morphology during the wound healing revealed that STS and NaHS-treated cells lost the typical elongated shape of VSMCs, as measured through the area, Feret diameter and circularity of the cells (Figure 5c). Of note, 15 mM STS did not induce VMSC apoptosis after 48 h, while 40 mM STS increased apoptosis levels to 5%. 80 mM STS for 48 h raised apoptosis levels to 10% (Figure S10).

### STS does not significantly impact VSMC metabolism

We previously showed that H_2_S inhibits the mitochondrial metabolism while increasing glycolysis in endothelial cells.^43^ To assess the impact of STS on VSMC metabolism, Seahorse experiments were performed on VSMCs treated for 4 or 24 h with STS. Surprisingly, STS did not inhibit mitochondrial respiration in a mitochondrial stress test after 4 h of pre-treatment, while NaHS had a slight effect on basal respiration and ATP production (Figure S11a). After 24 h of treatment, both STS and NaHS increased respiration mildly (Figure S11b). We also performed a glycolysis stress test to evaluate the metabolic flexibility of VSMCs (Figure S11c). After 24 h of pre-treatment, neither STS nor NaHS impacted glycolysis. Of note, VSMCs had a null glycolytic reserve, i.e. glycolysis did not increase upon inhibition of mitochondrial respiration with oligomycin. Further analysis of oxphos proteins by western blot showed no effect of a 24 h STS treatment on key mitochondrial enzymes (Figure S12). Overall, STS effect on mitochondrial respiration and glycolysis cannot explain the reduced proliferation and migration observed.

### STS interferes with microtubules organization

**Figure 6:**
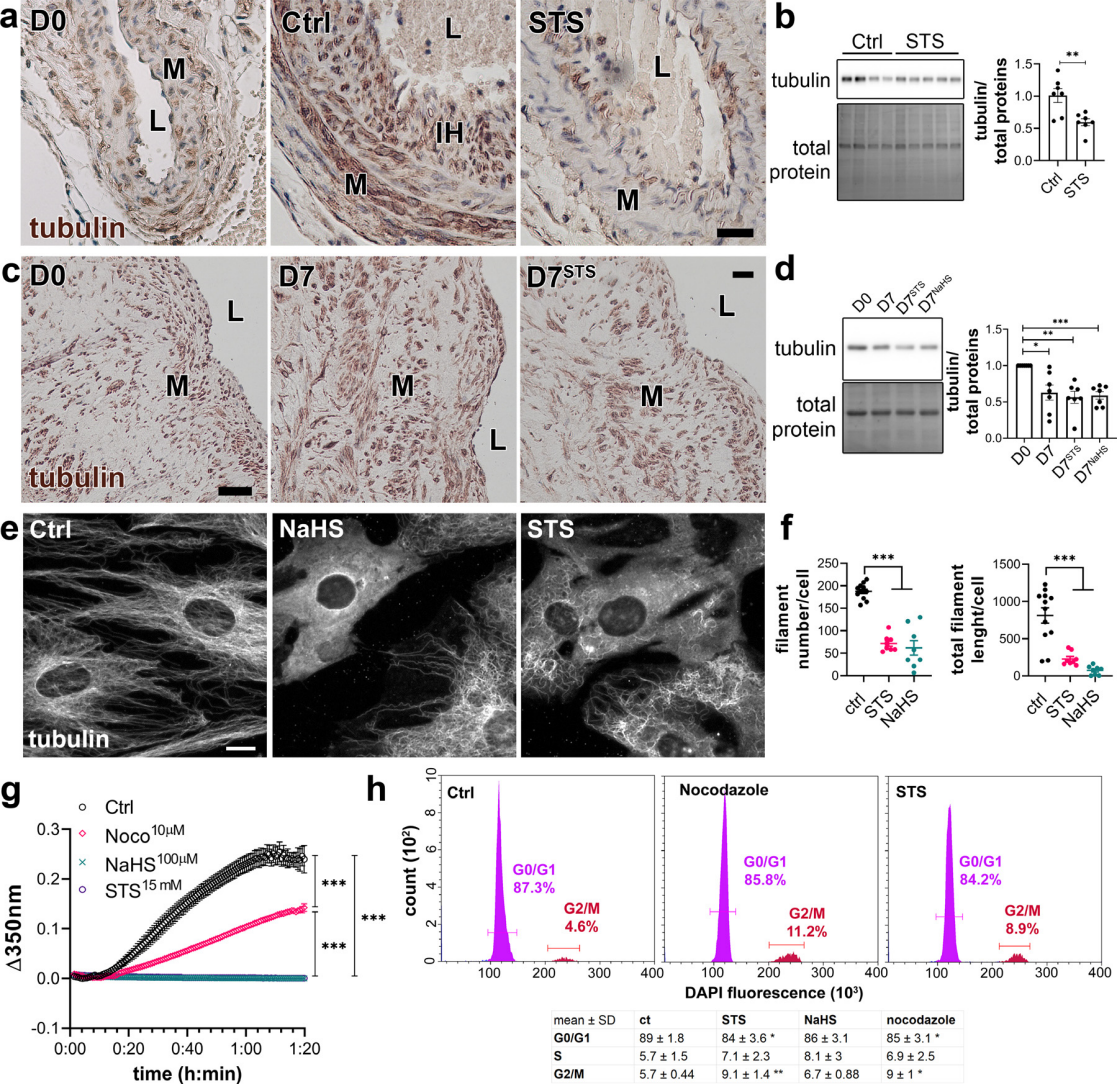
**STS inhibits microtubule polymerization in VSMC.** (a) α-tubulin immunolabelling in carotids of native (D0) or CAS-operated mice treated or not (Ctrl) with STS 4 g/L. L=Lumen; M= Media; IH= Intimal Hyperplasia. Images are representative of 5 to 8 mice per group. Scale bar 20 µm (b) WB analysis of α-tubulin over total protein in aortas of mice treated or not (Ctrl) with STS 4 g/L for 7 days. Data are scatter plots of 7 mice per groups with mean ± SEM with **p < .01, as determined by one-way ANOVA with Tukey's multiple comparisons tests. (c) α-tubulin immunolabelling in human vein segments kept or not (D0) in culture in presence or not (Ctrl) of 15 mM STS for 7 days. Scale bar 40 µm. L=Lumen; M=Media. Images are representative of 5 different veins. (d) WB analysis of tubulin over total protein in human vein segments kept or not (D0) in culture in presence or not (Ctrl) of 15 mM STS or 100 µm NaHS for 7 days. *p < .05, **p < .01, ***p < .001, as determined by repeated measures one-way ANOVA from 7 different veins with Dunnett's multiple comparisons tests. (e) α-tubulin immunofluorescent staining in VSMC exposed or not to 15 mM STS or 100 µm NaHS for 8 h. Images are representative of 5 independent experiments. Bar scale 10 µm. (f) Quantitative assessment of microtubule filaments immunostaining per cell. Data are representative of 3 independent experiments, 3 to 4 images per experiment per condition. ***p < .001 as determined by one-way ANOVA with Tukey's multiple comparisons tests. (g) *In vitro* tubulin polymerization assay in presence or not (Ctrl) of 15 mM STS, 100 µm NaHS or 10 µm Nocodazole. Data are mean ± SEM of 3 independent experiments. (h) Flow cytometry analysis of cell cycle (DNA content) using DAPI-stained VSMC treated or not (Ctrl) for 48 h with 15 mM STS or 10 nm Nocodazole. *Upper panel*: representative histograms; *lower panel*: table with mean ± SD of 5 independent experiments. *p < .05, **p < .01 as determined by one-way ANOVA with Dunnett's multiple comparisons tests.

Given the impact of STS on cell morphology, we examined the effect of STS on the cytoskeleton. α-tubulin levels were increased in the carotid wall of CAS-operated mice, which were reduced by STS, as demonstrated by immunohistochemistry (Figure 6a). α-tubulin levels were also decreased in the native aorta of mice treated with STS for 7 days (Figure 6b). On the other hand, STS did not impact tubulin staining in native carotids of WT and LDLR^−/−^ mice (Figure S13a). STS treatment also had no effect on tubulin levels in the liver and kidney (Figure S13b). In *ex vivo* vein segments, total α-tubulin levels were decreased after 7 days of STS or NaHS treatment (Figure 6c, d). Looking further at α-tubulin by immunofluorescent staining showed a loss in microtubule in VSMCs treated with STS or NaHS for 8 h (Figure 6 e, f). To study the effect of H_2_S on microtubule formation, an *in vitro* tubulin polymerization assay was performed in presence of 15 mM STS, 100 µm NaHS or 10 µM Nocodazole, an inhibitor of microtubule assembly. As expected, Nocodazole slowed down microtubule assembly as compared to the control. Surprisingly, both NaHS and STS fully blocked microtubule assembly in this assay (Figure 6g). Further studies of the cell cycle in VSMCs revealed that 48 h of treatment with STS or Nocodazole resulted in accumulation of cells in G2/M phase (Figure 6h).

## Discussion

Despite decades of research and the advent of drug-eluting stents (DES) and drug-coated balloons (DCB), intimal hyperplasia (IH) remains one of the major limitations in the long-term success of revascularization. All current strategies based on the use of DES and DCB limit VSMC proliferation and IH, but they also affect re-endothelisation, limiting their long-term efficacy and prolonging the need for anti-thrombotic. Moreover, recent controversies regarding the long-term safety of paclitaxel-releasing devices^6^, ^7^, ^8^, ^9^, ^10^, ^11^, ^12^, ^13^ advocate for the development of new therapies to limit IH. These new strategies should focus on inhibiting VSMC proliferation while promoting endothelium recovery. In that regard, the gasotransmitter H_2_S possesses interesting properties. Here, we demonstrate that exogenous sulfur supplementation in the form of STS limits IH development *in vivo* following mouse carotid artery stenosis. Furthermore, STS reduced apoptosis, vessel remodelling and collagen deposition, along with IH development in our *ex vivo* model of IH in human vein segments. We propose that STS limits IH by interfering with the microtubule dynamics, thus VSMC proliferation and migration.

An ever increasing number of studies document the protective effects of H_2_S against cardiovascular diseases,^44^ including studies showing that H_2_S reduces IH in preclinical models.^19^, ^20^, ^21^^,^^45^ The administration of H_2_S in those studies relies on soluble sulfide salts such as NaHS with narrow therapeutical range due to fast and uncontrolled release. Thiosulfate is an intermediate of sulfur metabolism shown to release H_2_S *in vivo* through non-enzymatic and enzymatic mechanisms.^25^^,^^26^^,^^46^^,^^47^ Importantly, STS is clinically approved and safe in gram quantities in humans. Although STS yields no detectable H_2_S or polysulfide *in vitro*, we observed increased circulating and liver levels of polysulfides in mice, as well as increased protein persulfidation in VSMCs. We further showed that STS treatment rescues Cse^−/−^ with impaired endogenous H_2_S production from increased IH. This is in line with a previous study showing that NaHS rescues increased IH in a model of carotid ligation in another Cse^−/−^ mouse line.^21^ Overall, STS has protective effects against IH similar to the H_2_S salt NaHS and several other “classical” H_2_S donors, but holds much higher translational potential.

Mechanistically, we first observed that STS reduces cell apoptosis and matrix deposition in our *ex vivo* model of human vein segments. This anti-apoptotic effect of STS and NaHS is in line with known anti-apoptotic effects of H_2_S.^44^ STS also reduces IH *in vivo* following carotid artery stenosis. In this model, matrix deposition plays little role in the formation of IH, which relies mostly on VSMC proliferation.^14^ Therefore, although ECM and especially collagen deposition are major features of IH in humans,^48^^,^^49^ reduced apoptosis and matrix deposition is not sufficient to fully explain the protection afforded by STS in carotids *in vivo*.

STS, similarly to the H_2_S donor NaHS, inhibits VMSC proliferation in the context of IH *in vivo* in mouse stenotic carotids, *ex vivo* in human vein segments, and *in vitro* in primary human VSMCs. These findings are in line with previous studies demonstrating that “classical” H_2_S donors decrease VSMCs proliferation in pre-clinical models.^20^^,^^22^^,^^50^ In mouse VSMC, exogenous H_2_S has been proposed to promote cell cycle arrest,^16^ and regulate the Mitogen-activated protein kinases (MAPK) pathway^19^ and Insulin-like Growth Factor (IGF-1) response.^51^ Regarding mouse VSMC migration, H_2_S may limit α5β1-integrin and matrix metalloproteinase-2 (MMP2) expression, preventing migration and ECMs degradation.^16^^,^^21^ In this study, we further document that STS and NaHS disrupt the formation of microtubules in human VSMCs *in vitro*. Our findings are in line with previous studies showing that Diallyl trisulfide, a polysulfide donor, inhibits microtubule polymerization to block human colon cancer cell proliferation.^52^ NaHS also depolymerizes microtubules within Aspergillus nidulans biofilms.^53^ The α/β tubulin dimer has 20 highly conserved cysteine residues, which have been shown to regulate microtubule formation and dynamics.^54^ In particular, thiol−disulfide exchanges in intra-chain disulfide bonds have been proposed to play a key role in microtubule assembly.^55^ Several high throughput studies of post-translational modification of protein cysteinyl thiols (-SH) to persulfides (-SSH) demonstrated that cysteine residues in α- and β-tubulin are persulfidated in response to H_2_S donors in various cell types.^56^^,^^57^ Given the prominent role of cytoskeleton dynamics and remodelling during mitosis and cell migration, we propose that STS/H_2_S-driven microtubule depolymerisation, secondary to cysteine persulfidation, contributes to cell cycle arrest and reduces migration in VSMC.

Our findings suggest that STS holds strong translational potential to limit restenosis following vascular surgeries. The dosage of STS used in this study is comparable to previous experimental studies using oral administration at 0.5 to 2 g/K g/day,^25^^,^^26^^,^^46^^,^^47^ and we did not observe any adverse effect of the 30-days treatment with STS on blood chemistry and plasmatic markers of kidney, liver and heart function. In humans, 12.5 and 25  g of STS have been infused without adverse effects^58^ and short-term treatment with i.v. STS is safely used in patients for the treatment of calciphylaxis.^24^ Of note, the pathophysiology of calciphylaxis, also known as calcific uremic arteriopathy (CUA), is caused by oxidative stress and inflammation, which promote endothelial dysfunction, leading to medial remodelling, inflammation, fibrosis and VSMC apoptosis and differentiation into bone forming osteoblast-like cells. Although the main effect of STS on CUA is via formation of highly soluble calcium thiosulfate complexes, our data supports the use of STS in the treatment of calciphylaxis. Plus, STS infusions have been shown to increase distal cutaneous blood flow, which could be beneficial in the context of vascular occlusive disease.

Here, we propose that STS treatment results in persulfidation of cysteine residues in the tubulin proteins, which lead to microtubule depolymerisation. However, further studies are required to test this hypothesis and demonstrate the STS-induced persulfidation of tubulin cysteine residues. In addition, although we show *in vitro* that H_2_S directly affect microtubule formation in a cell-free environment, other proteins involved in the microtubules dynamics *in vivo* may also be modified by H_2_S, and contribute to the effect of STS on microtubule polymerisation and cell proliferation. This effect on microtubules could lead to side effects similar to microtubule targeting agents such as nocodazole or colchicine and paclitaxel, which are widely used in the treatment of various cancers. However, unlike chemotherapeutical agents, the effect of STS/H_2_S should be reversible, which should reduce side effects. That said, additional experiments in larger animals are required to better assess haematological, gastrointestinal and neurological toxicities of long-term treatment with STS. In this study, STS was administered in the water bottle. However, given the low and variable bioavailability of oral STS, only intravenous STS should be prescribed.^58^ Moving forward, further studies in large animals are required to validate the therapeutical potential and setup the i.v. dose of STS required in a model of restenosis. Case reports and case series suggest that i.v. STS administration is safe, even for relatively long periods of time.^59^^,^^60^ However, randomised controlled trials testing long-term administration of STS are lacking,^60^ and the long-term safety and effects of STS administration should be further explored.

In summary, under the conditions of these experiments, STS, a FDA-approved compound, limits IH development *in vivo* in a model of arterial restenosis and *ex vivo* model in human veins. STS most likely acts by increasing H_2_S bioavailability, which inhibits cell apoptosis and matrix deposition, as well as VSMC proliferation and migration via microtubules depolymerisation.

## Contributors

FA, AL and SD designed the study. FA, DM, MMA, ML and SU performed the experiments. FA, DM, MMA, ML and SD analysed the data. FA, DM, MMA, AL and SD wrote the manuscript. FA, AL, SD and DM revised the manuscript.

### Data sharing statement

The data that support the findings of this study are available from the corresponding author, Florent Allagnat, upon request.

## Declaration of interests

The authors declare no competing interests.

## Appendix Supplementary materials

Image, application 1

Image, application 2

Image, application 3
