# Supplementary material for: Sodium thiosulfate acts as a hydrogen sulfide mimetic to prevent intimal hyperplasia via inhibition of tubulin polymerisation
Source: eBioMedicine. 2022 Mar 22;78:103954. doi: 10.1016/j.ebiom.2022.103954 (PMC8941337; doi:10.1016/j.ebiom.2022.103954)
Supplement: Supplementary file 1 [file mmc1.docx]

DATA SUPPLEMENT

**Sodium Thiosulfate acts as an H_2_S mimetic to prevent intimal hyperplasia via inhibition of tubulin polymerization.**

Diane Macabrey^1,2^, Alban Longchamp^1,2^, Michael R. MacArthur^3^, Martine Lambelet^1,2^, Severine Urfer^1,2^, Sebastien Deglise^1,2*^ and Florent Allagnat^1,2*^

^1^ Department of Vascular Surgery, Lausanne University Hospital, Switzerland

^2^ Department of Biomedical Sciences, University of Lausanne, Switzerland

^3^ Department of Health Sciences and Technology, Swiss Federal Institute of Technology (ETH) Zurich, Zurich, Switzerland.

*These authors contributed equally to this work as senior authors.

**Supplementary Methods**

## Apoptosis assay

Apoptosis TUNEL assay was performed using the DeadEnd^TM^ Fluorometric TUNEL system kit on frozen sections of human vein segments. Immunofluorescent staining was performed according to the manufacturer’s instruction. Apoptotic nuclei were automatically detected using the ImageJ software and normalized to the total number of DAPI-positive nuclei. *In vitro* VSMC apoptosis was determined by Hoechst/Propidium Iodide staining of live VSMCs and manually counted by two independent blinded experimenters (1).

## Seahorse

Glycolysis and Mitochondrial stress tests were performed on confluent VSMCs according to the manufacturer’s kits (Seahorse XF Glycolysis Stress Test Kit and Seahorse XF Cell Mito Stress Test Kit) and protocol (Agilent Technologies). Cells were treated for 4 or 24 hours with NaHS or STS before the seahorse experiments. Data were analysed using the Seahorse Wave Desktop Software (Agilent Technologies).

## Blood analyses

Analyses were performed on blood from mice treated for 3 weeks with 0.5g/Kg/day sodium thiosulfate. Blood chemistry (pH, HCO3, Na, K and Ca) analyses were performed on whole blood (95µL) immediately upon cardiac puncture using an i-STAT apparatus equipped with CG8^+^ cartridges. Levels of Urea, CK, CK-MB, ASAT and ALAT were measured in heparinised plasma in a Cobas 8000 (Roche Diagnostics; Lausanne University Hospital).

## Systolic blood pressure measurement

Systolic blood pressure (SBP) was monitored daily by non-invasive plethysmography tail cuff method (BP-2000, Visitech Systems Inc.) on conscious mice (2).

**Supplementary Table S1: Antibodies**

| **Target antigen** | **Vendor** | **Catalog #** | **Working**  **concentration** | **RRID** |
| --- | --- | --- | --- | --- |
| **Collagen III** | Abcam | ab7778 | 1/100 (IHC-Fz) | AB_306066 |
| **Bax** | Santa Cruz | Sc-526 | 1/500 (WB) | AB_2064668 |
| **Bcl2** | Santa Cruz | Sc-492 | 1/500 (WB) | AB_2064290 |
| **Goat anti-Rabbit IgG Secondary Antibody, Alexa Fluor 680** | Thermo Fisher Scientific | A21109 | 1/250 (ICC) | AB_2535758 |
| **α-Tubulin** | Sigma-Aldrich | T6074 | 1/10000 (WB)  1/1000 (IHC-P) | AB_477582 |
| **Anti-Rabbit HRPO** | Thermo Fisher Scientific | 31460 | 1/20000 (WB) | AB_228341 |
| **Anti-mouse HRPO** | Jackson ImmunoResearch Labs | 115-035-146 | 1/15000 (WB) | AB_2307392 |
| **BrdU** | BD Biosciences | 555627 | 1/200 (ICC) | AB_10015222 |
| **P4HA1** | Proteintech | 12658-1-AP | 1/1000 (IHC-P) | AB_2283162 |
| **PCNA** | Dako (Now Agilent) | M0879 | 1/100 (IHC-P) | AB_2160651 |
| **Cleaved Caspase 3** | Cell Signaling Technology | 9661 | 1/200 (IHC-P) | AB_2341188 |
| **Total OXPHOS Human WB Antibody Cocktail** | Abcam | ab110411 | 1/5000 (WB) | AB_2756818 |
| **CSE** | Proteintech | 12217-1-AP | 1/1000 (WB) | AB_2087497 |
| **CBS** | Santa Cruz | sc-133154 | 1/1000 (WB) | AB_2244094 |
| **3-MST** | Novus | NBP1-82617 | 1/2000 (WB) | AB_11014969 |

**Supplementary Table S2: Reagents**

| **Kit/product** | **Vendor** | **Catalog #** | **link** |
| --- | --- | --- | --- |
| Sodium Thiosulfate | Hänseler AG | 06-6688-01 | https://www.reactolab.ch/boutique/hanseler/sodium-thiosulfate-500-gr/ |
| NaHS | Sigma-Aldrich | 161527 | <https://www.sigmaaldrich.com/catalog/product/sigald/161527?lang=fr&region=CH&cm_sp=Insite-_-caSrpResults_srpRecs_srpModel_16721-80-5-_-srpRecs3-1> |
| GYY4137 | Sigma-Aldrich | SML0100 | https://www.sigmaaldrich.com/CH/en/product/sigma/sml0100 |
| Diallyl Trisulfide (DATS) | Cayman Chemical | 10 012577 | https://www.caymanchem.com/product/10012577/diallyl-trisulfide |
| H_2_S Donor 5a. | Cayman Chemical | 11238 | https://www.caymanchem.com/product/11238/h2s-donor-5a |
| Sodium trisulfide (Na_2_S_3_) | SulfoBiotics | SB03-10 | https://www.dojindo.eu.com/store/p/859-SulfoBiotics-Sodium-trisulfide-Na2S3.aspx |
| Nocodazole | Sigma-Aldrich | M1404 | https://www.sigmaaldrich.com/CH/en/product/sigma/m1404 |
| *In Vitro* Tubulin Polymerization Assay Kit | Sigma-Aldrich | 17-10194 |  |
| DeadEnd Fluorometric TUNEL system | Promega | G3250 | <https://ch.promega.com/products/cell-health-assays/apoptosis-assays/deadend-fluorometric-tunel-system/?catNum=G3250> |
| Immobilon Western Chemiluminescent HRP Substate | Millipore | WBKLS0050 | [https://www.merckmillipore.com/CH/de/product/Immobilon-Western-Chemiluminescent-HRP-Substrate,MM_NF-WBKLS0050](https://www.merckmillipore.com/CH/de/product/Immobilon-Western-Chemiluminescent-HRP-Substrate,MM_NF-WBKLS0050?ReferrerURL=https%3A%2F%2Fwww.google.com%2F) |
| Immobilon-P transfer membrane | Millipore | IPVH00010 | <https://www.merckmillipore.com/CH/de/product/Immobilon-P-PVDF-Membrane,MM_NF-IPVH00010> |
| EnVision®  + Dual Link System-HRP (DAB+) | DAKO (now Agilent) | K4065 | <https://www.agilent.com/cs/library/packageinsert/public/PD04048EFG_01.pdf> |
| Antifade Mounting Medium with DAPI | Vectashield | H-1200 | <https://vectorlabs.com/products/mounting/vectashield-with-dapi> |
| Pierce reversible protein Stain Kit for PDGF membranes | Thermo Fisher Scientific | 24585 | <https://www.thermofisher.com/order/catalog/product/24585#/24585> |
| Seahorse XF Glycolysis Stress Test Kit | Agilent | 103020-100 | https://www.agilent.com/store/productDetail.jsp?catalogId=103020-100&catId=SubCat2ECS_897073 |
| Seahorse XF Cell Mito Stress Test Kit | Agilent | 103015-100 | https://www.agilent.com/store/en_US/Prod-103015-100/103015-100 |
| DirectPCR Lysis Reagent (Ear) | Viagen | 402-E | http://www.viagenbiotech.com/index.php/directpcr-lysis-reagents/tail/500-mouse-tails-100-ml.html |
| Proteinase K | Qiagen, | 1122470 | https://www.qiagen.com/us/products/discovery-and-translational-research/lab-essentials/enzymes/qiagen-protease-and-proteinase-k/?catno=19131 |
| Platinum™ *Taq* DNA Polymerase | Invitrogen | 10966-026 | https://www.thermofisher.com/order/catalog/product/10966026 |
| RPMI-1640 Glutamax I | Gibco | 61870-010 | <https://www.thermofisher.com/RPMI> |
| Gelatin type B | Sigma-Aldrich | G9391 | [G9391 Sigma](https://www.sigmaaldrich.com/catalog/product/sigma/g9391?lang=fr&region=CH&gclid=EAIaIQobChMI1aaDp4HZ6QIVhrHtCh1rbQ7ZEAAYASAAEgJWf_D_BwE) |
| Tripure | Roche | 11667157001 | [Roche_tripure](https://www.sigmaaldrich.com/catalog/product/roche/tripurero?lang=fr&region=CH) |
| DAz-2 | Cayman Chemicals | 13382 | [www.caymanchem.com/product/13382](https://www.caymanchem.com/product/13382) |
| Cyanine 5.5 alkyne | Lumiprobe | C70B0 | https://www.lumiprobe.com/p/cy55-alkyne |
| 4-Chloro-7-Nitrobenzofurazan | Sigma Aldrich | 163260 | <https://www.sigmaaldrich.com/catalog/product/aldrich/163260?lang=fr&region=CH> |
| SF_7_-AM fluorescent probe | Sigma-Aldrich | 748110 | [Sigmaaldrich_748110](https://www.sigmaaldrich.com/catalog/product/aldrich/748110?lang=fr&region=CH) |
| SSP4 fluorescent probe | Dojindo Molecular Technologies | SB10 | https://www.dojindo.com/product/sulfobiotics-ssp4-sb10/ |
| Ketamin (Ketasol-100) | Gräub E.Dr.AG, Bern Switzerland | NA | <https://www.graeub.com/fr/products/product/ketasol-100/2054> |
| Xylasin (Rompun®), | Provet AG, Lyssach, Switzerland | NA | <http://www.provet.gr/en/animal-health/products/pharmaceuticals/veterinary-pharmaceuticals/rompun-inj.-sol./7-489> |
| Buprenorphine (Temgesic) | Reckitt Benckiser AG, Switzerland | NA | [Temgesic](https://pharmaceutical.network/search.aspx/xxxqsum/Temgesic%C2%AE) |
| Resorcine-Fuchsine Weigert | Waldeck | 2E-030 | <https://www.reactolab.ch/boutique/chroma/resorcin-fuchsine-weigert-solution-500-ml/> |
| Hematoxylin crist. | Merck | 1.04302.0025 | [Hematoxylin-cryst](https://www.merckmillipore.com/CH/de/product/Hematoxylin-cryst.-C.I.-75290,MDA_CHEM-104302?ReferrerURL=https%3A%2F%2Fwww.google.com%2F) |
| Acid Fuchsin | Sigma-Aldrich | F8129 | <https://www.sigmaaldrich.com/catalog/product/sigma/f8129?lang=fr&region=CH> |
| Picric acid | Sigma-Aldrich | 197378 | <https://www.sigmaaldrich.com/catalog/product/aldrich/197378?lang=fr&region=CH> |
| Neo-Clear | Merck | 1.09843.5000 | [Neo-Clear](https://www.merckmillipore.com/CH/de/product/Neo-Clear,MDA_CHEM-109843?ReferrerURL=https%3A%2F%2Fwww.google.com%2F) |
| Ethanol absolu | Merck | 1.00983.2500 | [Ethanol](https://www.merckmillipore.com/CH/de/product/Ethanol,MDA_CHEM-100983?ReferrerURL=https%3A%2F%2Fwww.google.com%2F) |
| Phosphate Buffered Saline (PBS) | Bichsel: | 100 0 324 | [https://www.bichsel.ch/](https://www.bichsel.ch/details-bichsel-news-146/items/brochure-bichsel.html) |
| i-STAT CG8+ CARTRIDGE | Abbot | 03P88-25 | https://www.globalpointofcare.abbott/en/product-details/apoc/istat-cg8plus-test-cartridge.html |
| Sodium dodecyl sulfate (SDS) | Promega | H5114 | [https://ch.promega.com/products/biochemicalssodium-dodecyl-sulfate](https://ch.promega.com/products/biochemicals-and-labware/biochemical-buffers-and-reagents/sodium-dodecyl-sulfate_-molecular-biology-grade-_sds_/?catNum=H5113) |
| Tween-20 | Applichem | A1389 | [https://www.applichem.com/tween](https://www.applichem.com/en/shop/product-detail/as/tweenreg-20-fuer-die-molekularbiologie/) |
| Triton x-100 | Sigma-Aldrich | T8787 | <https://www.sigmaaldrich.com/catalog/product/sigma/t8787?lang=fr&region=CH> |
| Bovine Serum albumin (BSA) | Applichem | A1391 | [https://www.applichem.comalbumin-fraktion-v](https://www.applichem.com/en/shop/product-detail/as/albumin-fraktion-v-ph-70/) |
| Recombinant Human PDGF-BB | PeproTech House | 100-14B | [recombinant-human-pdgf-bb](https://www.peprotech.com/fr/recombinant-human-pdgf-bb) |
| *DC*™ Protein Assay Kit I | Bio-Rad Laboratories | 5000111 | [Bio-rad dc-protein-assay](https://www.bio-rad.com/en-ch/product/dc-protein-assay?ID=22faf97a-6b8d-4763-8b97-3dc530dcab66) |
| Fast SYBR™ Green Master Mix | Applied Biosystems | 4385618 |  |
| PrimeScript RT Reagent Kit (Perfect Real Time) | Takara Bio | RR037B | https://www.takarabio.com/products/real-time-pcr/reverse-transcription-prior-to-qpcr/primescript-rt-reagent-kit |

**Supplementary Table S3: Blood panel**

| Mean (SD) | Ctrl (n=8) | STS (n=8) | *p* |
| --- | --- | --- | --- |
| pH | 6.99 (0.07) | 7.03 (0.05 | *0.23* |
| HCO3 (*mM)* | 21.52 (2.48) | 21.85 (1.24) | *0.74* |
| Na (*mM*) | 150.1 (2.85) | 147.5 (3.30) | *0.11* |
| K (*mM*) | 5.6 (0.63) | 4.8 (0.66) | *0.02* |
| Ca (*mM*) | 1.39 (0.06) | 1.35 (0.1) | *0.33* |
| Urea (*mg/dL*) | 68 (8.4) | 56 (10) | *0.04* |
| CK (*U/L*) | 177.4 (47) | 202.8 (50) | *0.71* |
| CK-MB (*U/L*) | 73.8 (8.5) | 86.6 (14.4) | *0.39* |
| ASAT (*U/L*) | 75.6 (8.8) | 82.4 (10.7) | *0.64* |
| ALAT (*U/L*) | 43.9 (8.1) | 39.5 (6.3) | *0.69* |

**
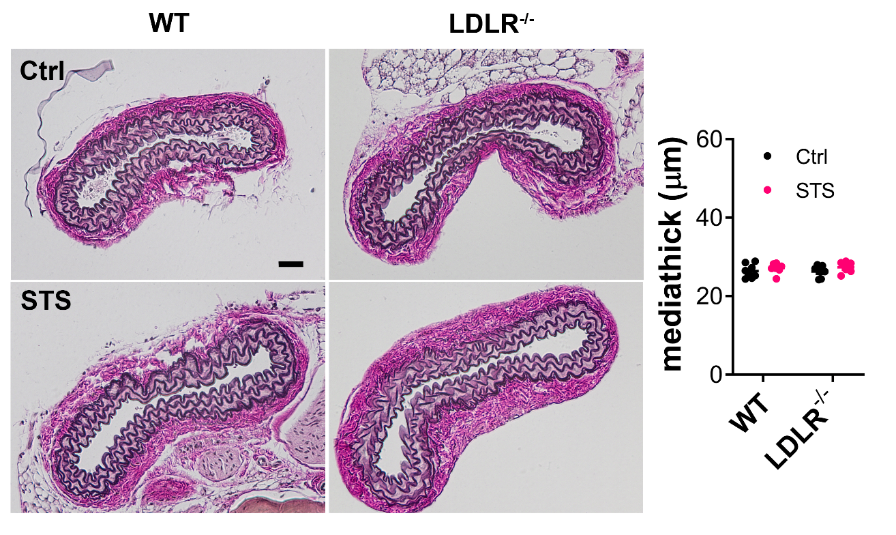
**

**Figure S1. STS does not impact media thickness in native carotids of WT and LDLR^-/-^ mice.**

WT and LDLR^-/-^ mice were treated for 28 days with 4 gr/L STS in the water bottle. Representative VGEL staining of carotid cross sections and morphometric measurements of media thickness. Data are mean±SEM of 6-8 animals per group.


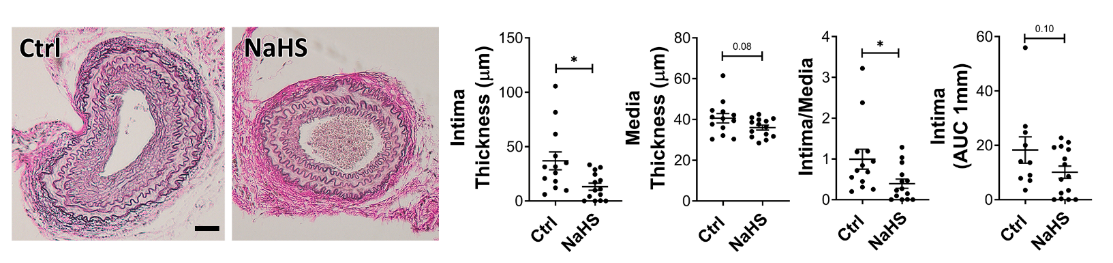


**Figure S2. NaHS decreases IH formation after carotid artery stenosis in mice**

WT mice were treated for 28 days post carotid artery surgery with 0.5 gr/L NaHS in the water bottle. Representative VGEL staining of operated left carotid cross sections and morphometric measurements of intima thickness, media thickness, intima over media ratio, and intima thickness AUC over 1mm from the ligation. Data are mean±SEM of 11 to 13 animals per group. *p<.05, as determined by ordinary one-way ANOVA with Dunnett's multiple comparisons.


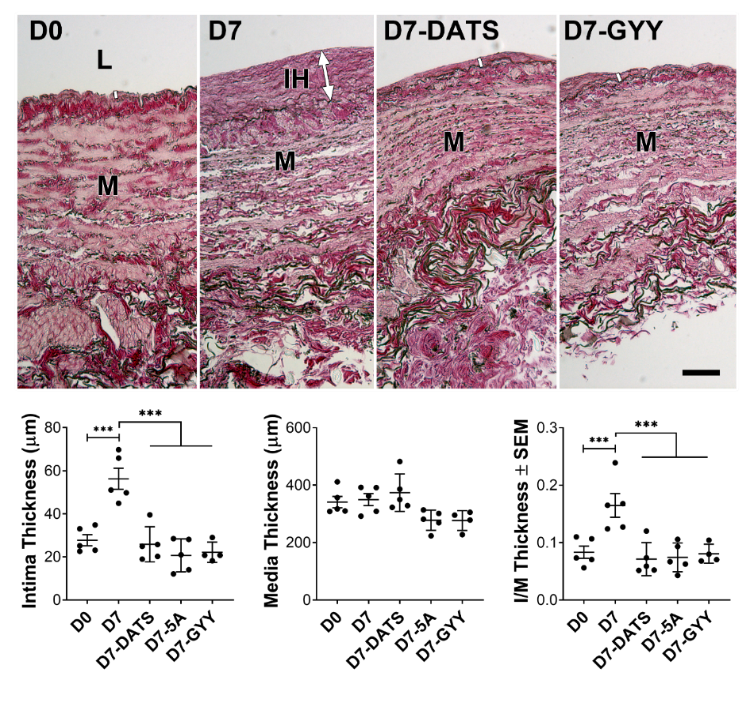


**Figure S3. H_2_S donors decrease IH formation in *ex vivo* vein segments.**

VGEL staining of human vein cross section and intima thickness, media thickness and intima over media ratio of freshly collected (D0) human vein segments after 7 days in static culture with or without (D7), diallyl trisulfide (DATS 200 µM), GYY4137 (GYY 200 µM) or Donor 5A (30 µM). Scale bar: 100 µm. Data are shown as mean±SEM of 5 different veins. *p<.05, **p<.01 as determined by repeated measures one-way ANOVA with Dunnett’s multiple comparisons. L=lumen; M= media; IH= intimal hyperplasia

**
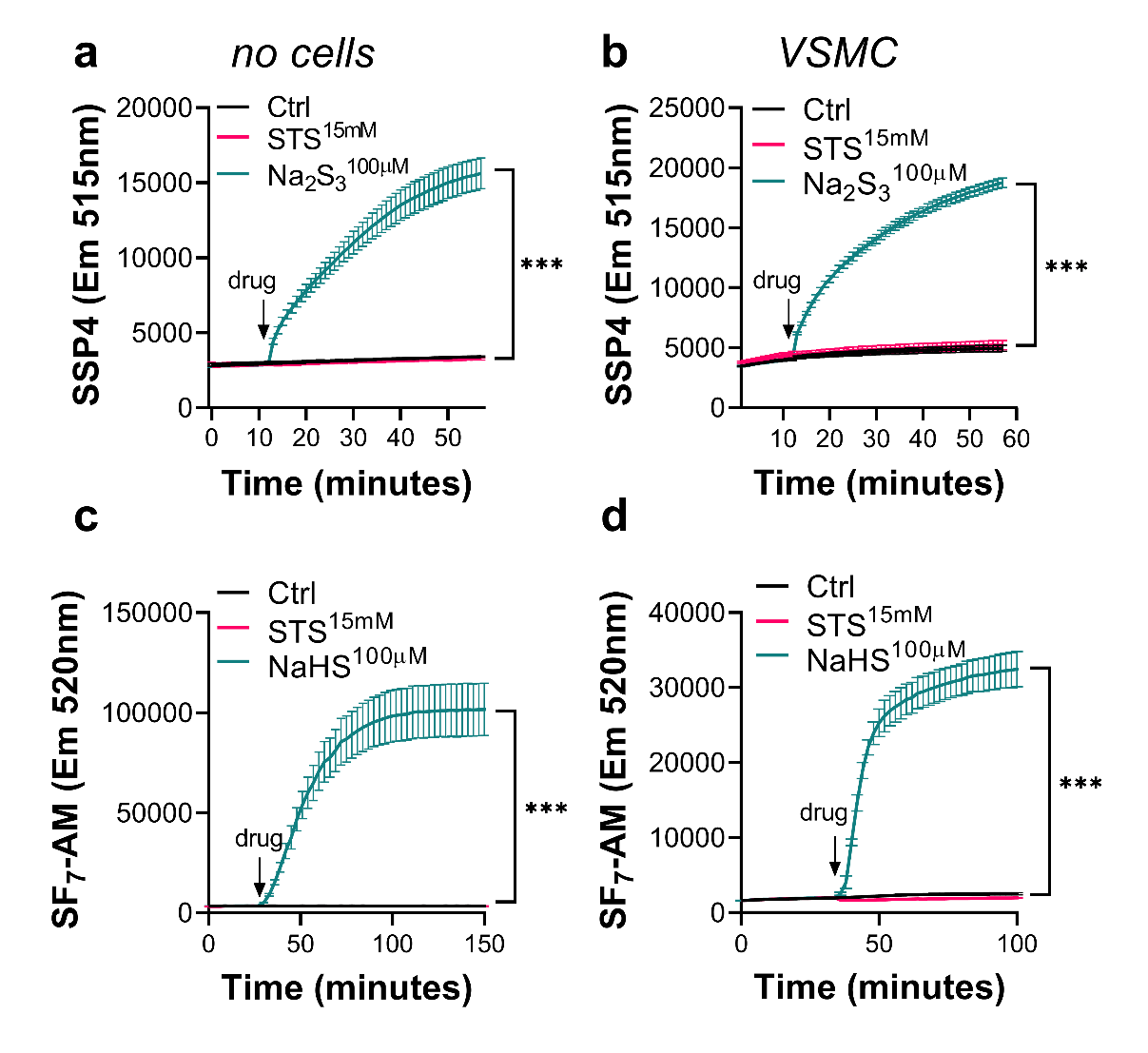
**

**Figure S4. STS does not release detectable amounts of H_2_S or polysulfides *in vitro***

**a-b)** Polysulfides release measured by the SSP4 probe in RPMI media without cells (**a**) or in presence of VSMC (**b**). **c-d)** H_2_S release measured by the SF_7_-AM probe in RPMI media without cells (**c**) or in presence of VSMC **(d)**. Data are mean±SEM of 4 independent experiments. ***p<.0001 as determined by repeated measure 2 way ANOVA with Tukey’s multiple comparisons.


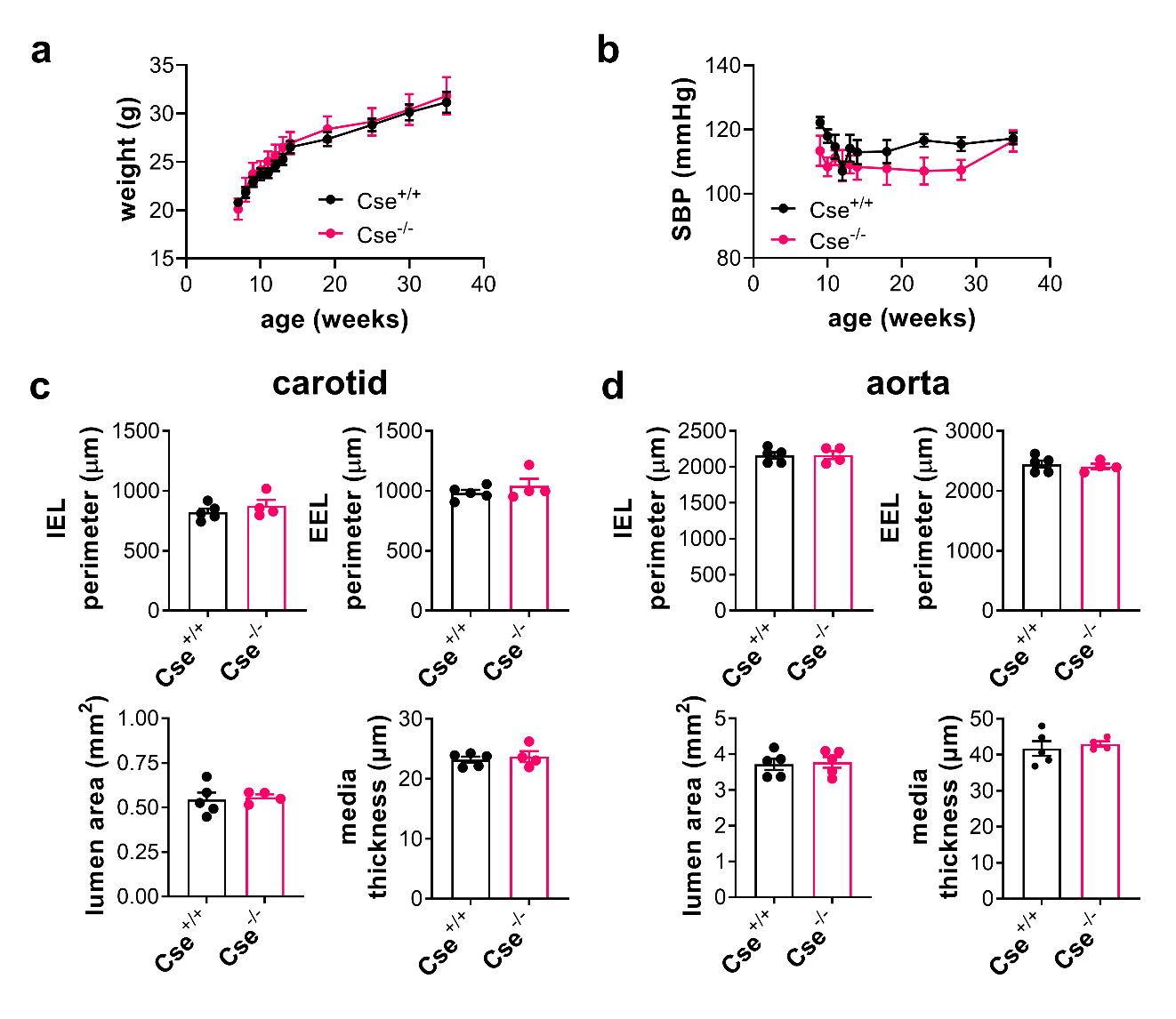


**Figure S5. Cse^-/-^ mice display no evident vascular phenotype**

**a)** Weight curves of Cse^-/-^ and WT (Cse^+/+^) littermates. **b**) Systolic Blood pressure (SBP) in Cse^-/-^ and WT (Cse^+/+^) littermates. **c-d**) Carotid and aorta histomorphometry in Cse^-/-^ and WT (Cse^+/+^), as measured on VGEL-stained cross section of carotid or aorta. Data are mean±SEM of 4 to 6 animals per group. IEL:internal elastic lamina; EEL:external elastic lamina.

**
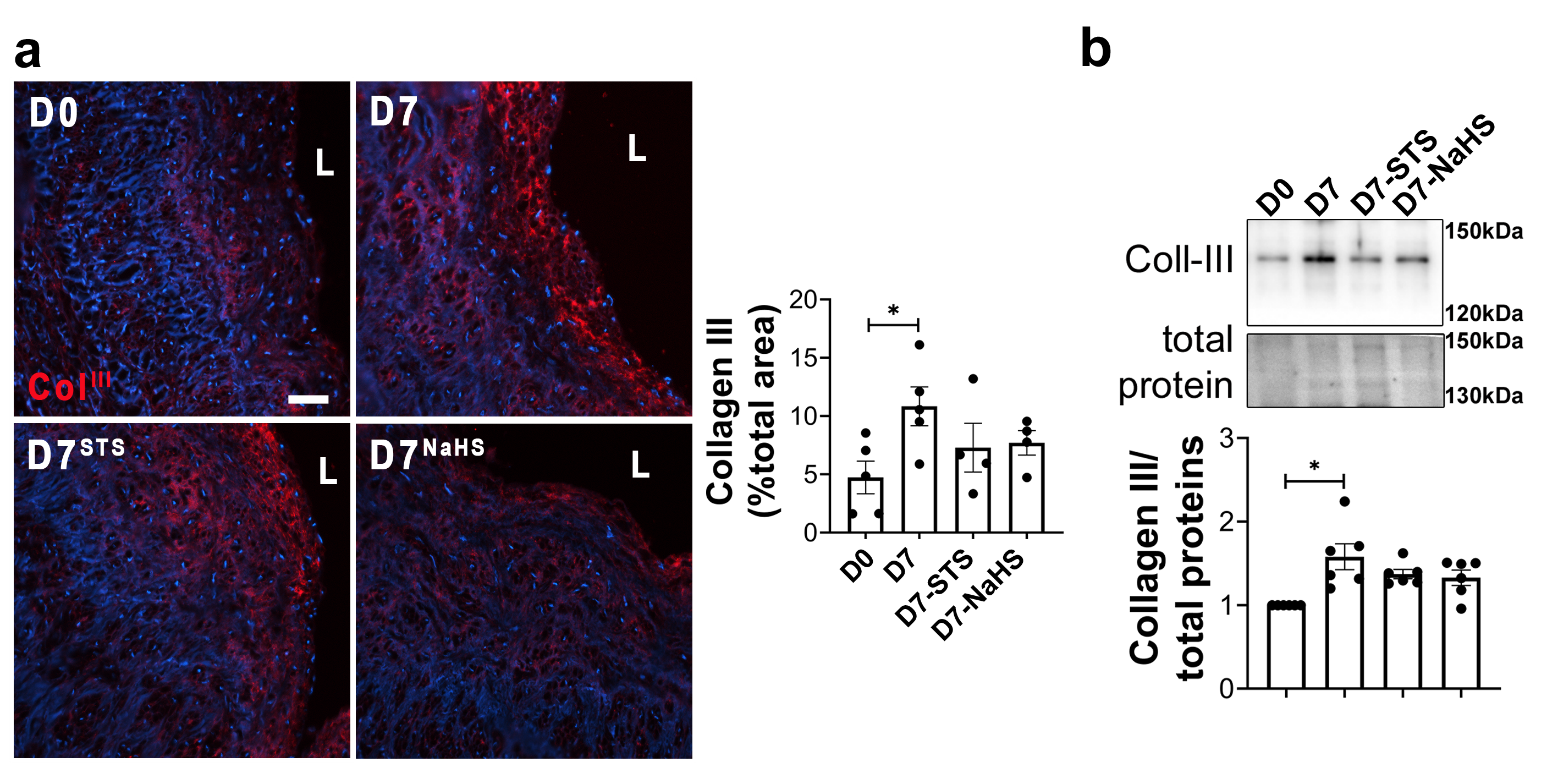
**

**Figure S6. STS and NaHS reduce collagen III accumulation in *ex vivo* vein segments**

**A**) *Left panel:* Representative collagen III immunofluorescent staining. Scale bar=50 µm. *Right panel:* Quantitative assessment of Collagen III immunofluorescent staining. Data are scatter plots of 5 different veins with mean±SEM. *p<.05 as determined by paired repeated measures one-way ANOVA with Dunnett’s multiple comparisons. **B)** Representative western blot of collagen III over total protein and quantitative assessment of 6 different human vein segments. Data are scatter plots with mean±SEM. *p<.05 as determined by paired repeated measures one-way ANOVA with Dunnett’s multiple comparisons.

**
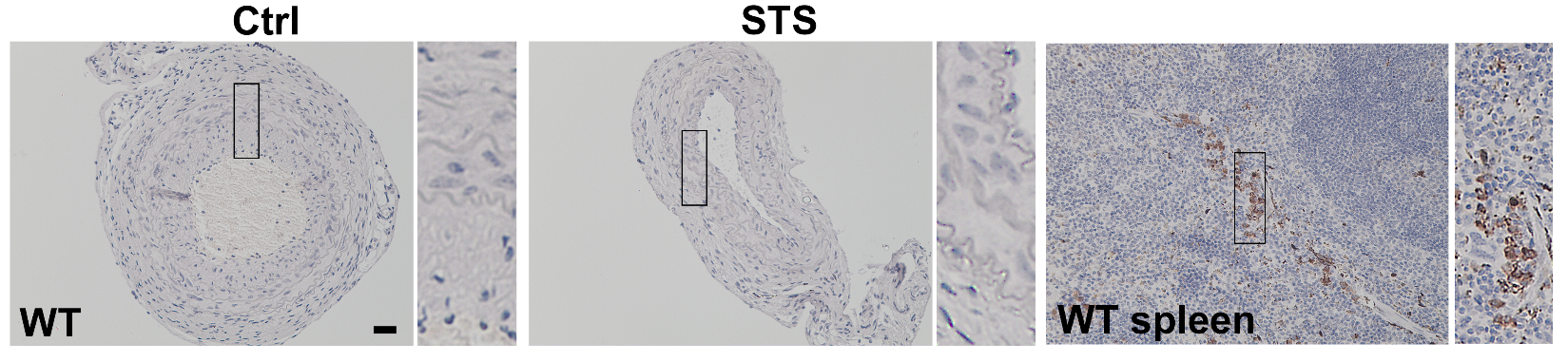
**

**Figure S7.** **STS does not induce cell apoptosis *in vivo* in mouse carotids**

Cleaved caspase 3 immunostaining (in brown) on CAS operated carotids in WT mice treated or not (Ctrl) with STS 4g/L for 28 days. Tissue was counterstained with hematoxylin to label nuclei (in blue). Spleen from a WT mouse was used as a positive control for Cleaved caspase 3 immunostaining. Scale bar 40 µm. Insets are 4 fold magnification of main images.


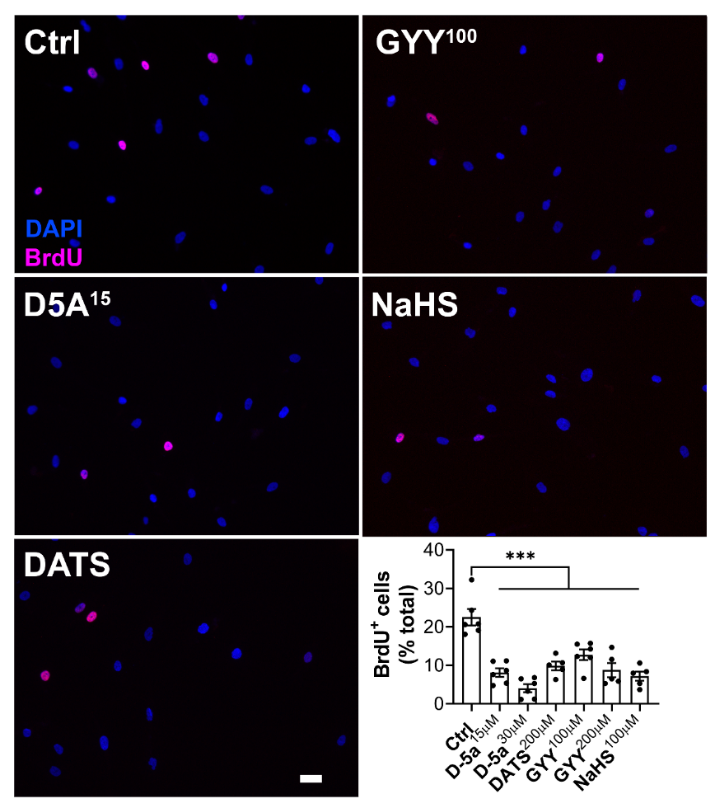


**Figure S8. H_2_S donors inhibit VSMC proliferation *in vitro***

VSMC proliferation as assessed by BrdU incorporation (pink) over total nuclei (blue) for 24 hours in presence or absence (Ctrl) of Diallyl trisulfide (DATS 200 µM) or GYY4137 (GYY 100 or 200 µM) or Donor 5A (D5A at 15 or 30 µM) and NaHS (100 µM). Scale bar: 25 µm. Data are mean±SEM of 6 independent experiments. ***p<.001 as determined by repeated measures ordinary one-way ANOVA with Dunnett's multiple comparisons.





**Figure S9. STS and NaHS inhibit VSMC migration**

VSMC migration in cells treated or not (Ctrl) with 15 mM STS or 100 µM NaHS in presence of 10 μg/mL Mitomycin C, as assessed by wound healing assay, expressed as the percentage of wound closure after 8 hours. Scale bar: 100 µm. Data are scatter plots with mean±SEM of 5 independent experiments in duplicates. ***p<.001 as determined by repeated measures one-way ANOVA with Dunnett’s multiple comparisons.


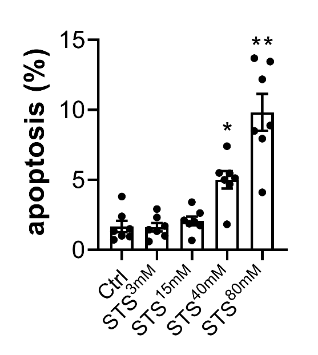


**Figure S10. STS is not cytotoxic at 15mM in VSMC**

VSMC apoptosis after 48 hours of cell culture in presence of increasing concentration of STS, as indicated. Data shown as mean ± SEM. *p<.05, **p<.01 as determined by repeated measures ordinary one-way ANOVA followed by Dunnett's multiple comparisons tests.

**
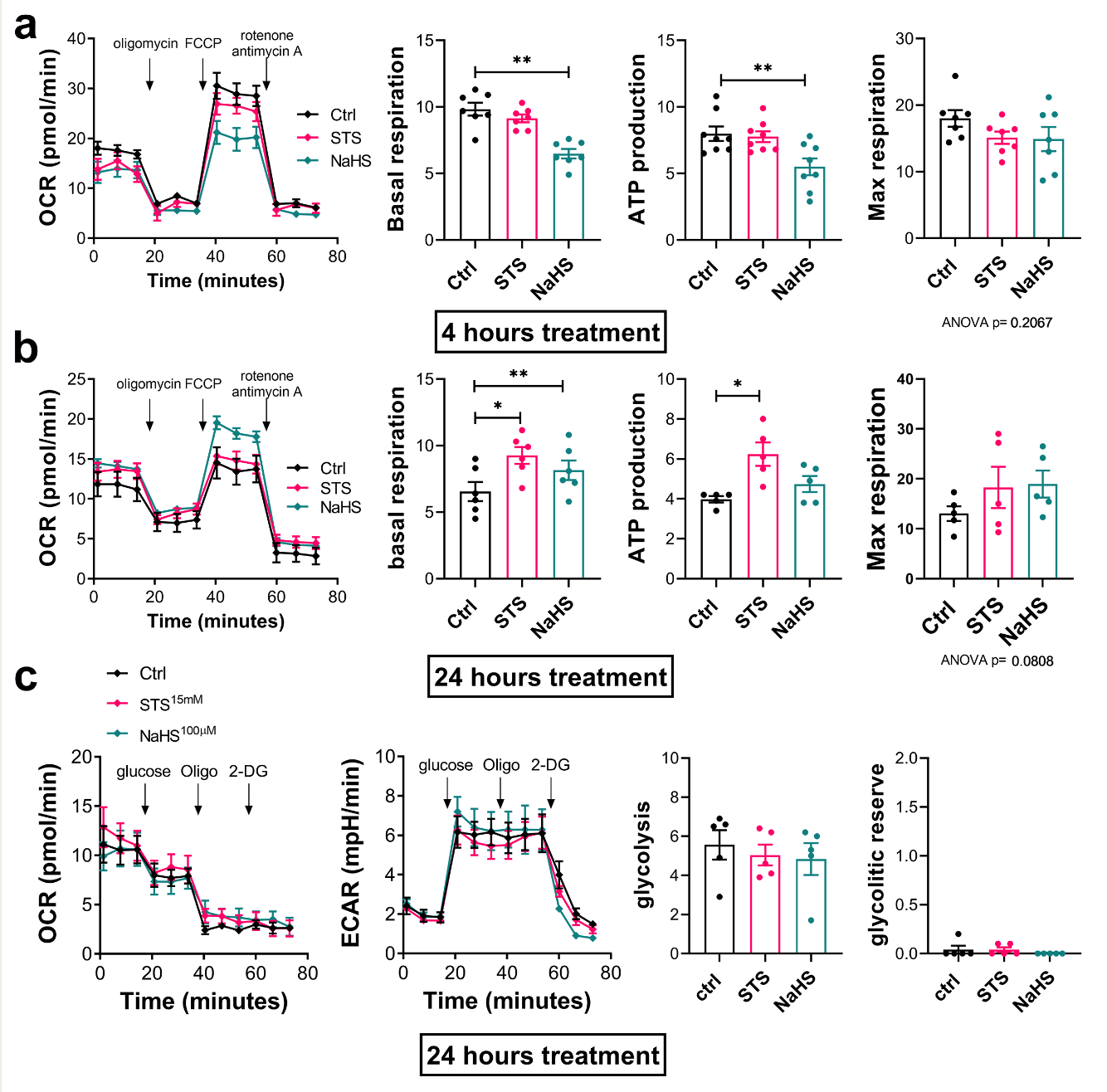
**

**Figure S11. STS does not significantly impact VSMC metabolism *in vitro***

**a-b)** Mito stress test assay in VSMC pre-treated or not (Ctrl) for 4h (**a**) or 24h (**b**) with 100 µM Nahs or 15 mM STS. **c)** Glucose stress test assay in VSMC pre-treated or not (Ctrl) 24h with 100 µM Nahs or 15 mM STS. Data are representative Seahorse traces and mean±SEM of 5-6 independent experiments. *p<.05, **p<.01 as determined by 2 way ANOVA with Tukey’s multiple comparisons.

**
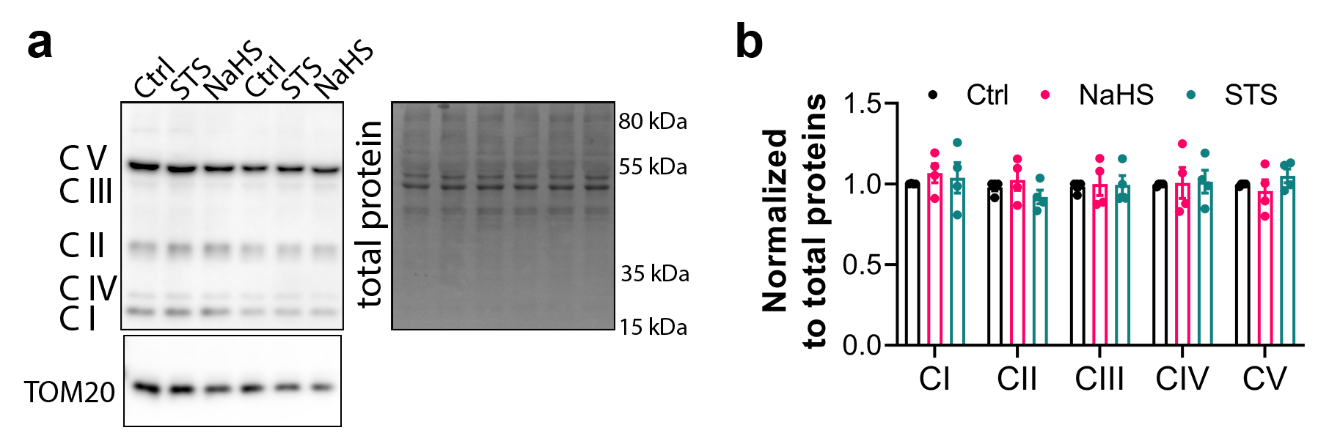
**

**Figure S12. STS does not significantly impact the mitochondrial respiratory chain in VSMC *in vitro***

VSMC were treated for 24 hours with 15 mM STS or 100 µM NaHS. Representative Western blot (**A**) and quantitative assessment of Oxphos complexes (**B**), normalized to total protein in 4 independent experiments.

**
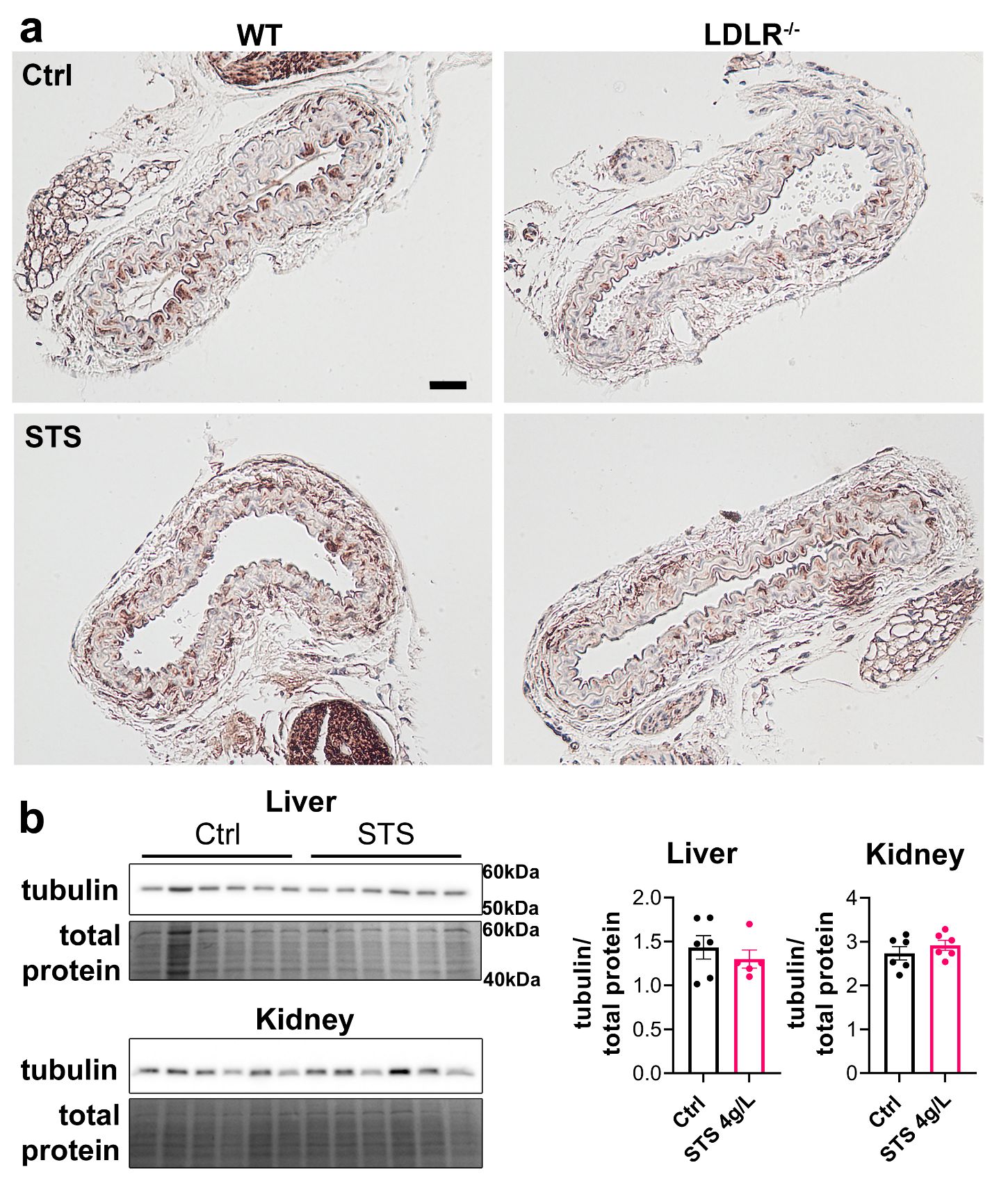
**

**Figure S13. STS treatment does not decrease tubulin in native carotids, liver and kidney of WT mice.**

**a)** WT and LDLR^-/-^ mice were treated for 28 days with 4 gr/L STS in the water bottle. Representative tubulin immunostaining of carotid cross sections. Data are mean±SEM of 4-6 animals per group. **b)** WT mice were treated for 28 days with 4 gr/L STS in the water bottle. Representative tubulin Western blotting and quantitative assessment of tubulin levels, normalized to total proteins, in the liver and kidney of 5-6 animals per group.

**References**

1. Allagnat F, Fukaya M, Nogueira TC, Delaroche D, Welsh N, Marselli L, et al. C/EBP homologous protein contributes to cytokine-induced pro-inflammatory responses and apoptosis in beta-cells. Cell Death Differ. 2012;19(11):1836-46.

2. Le Gal L, Alonso F, Wagner C, Germain S, Nardelli Haefliger D, Meda P, et al. Restoration of connexin 40 (Cx40) in Renin-producing cells reduces the hypertension of Cx40 null mice. Hypertension. 2014;63(6):1198-204.
