# Supplementary material for: Sodium thiosulfate acts as a hydrogen sulfide mimetic to prevent intimal hyperplasia via inhibition of tubulin polymerisation
Source: eBioMedicine. 2022 Mar 22;78:103954. doi: 10.1016/j.ebiom.2022.103954 (PMC8941337; doi:10.1016/j.ebiom.2022.103954)
Supplement: Supplementary file 2 [file mmc2.docx]

Caption for supplementary material

The supplementary material file contains supplementary methods related to the supplementary figures, as well as tables with detailed lists of the material and reagents used in this study.

It also contains the uncropped full blots (with labelling) for all Western blots that appear in the manuscript.
