# Supplementary figures and images for: Sodium thiosulfate acts as a hydrogen sulfide mimetic to prevent intimal hyperplasia via inhibition of tubulin polymerisation
Source: eBioMedicine. 2022 Mar 22;78:103954. doi: 10.1016/j.ebiom.2022.103954 (PMC8941337; doi:10.1016/j.ebiom.2022.103954)

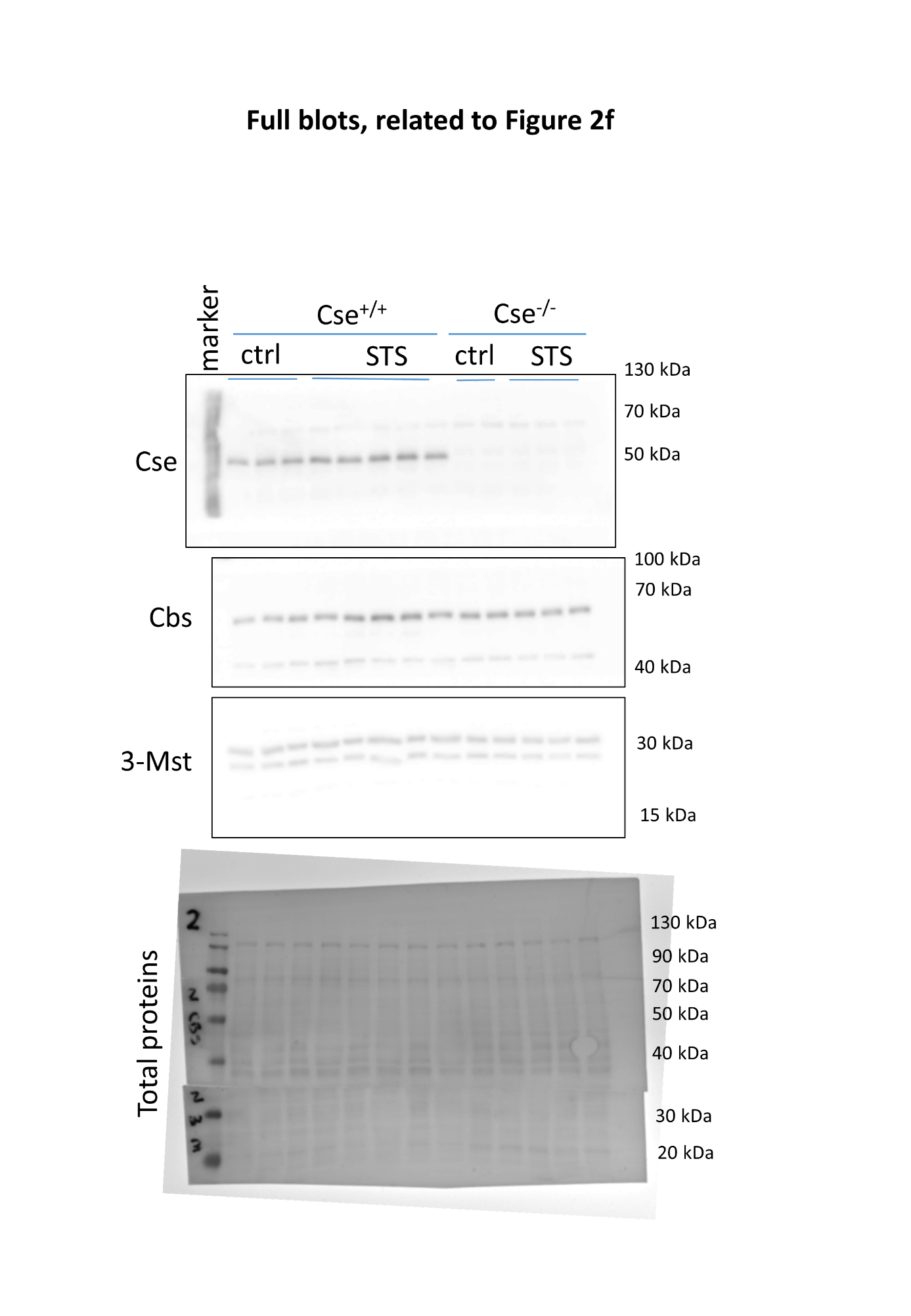

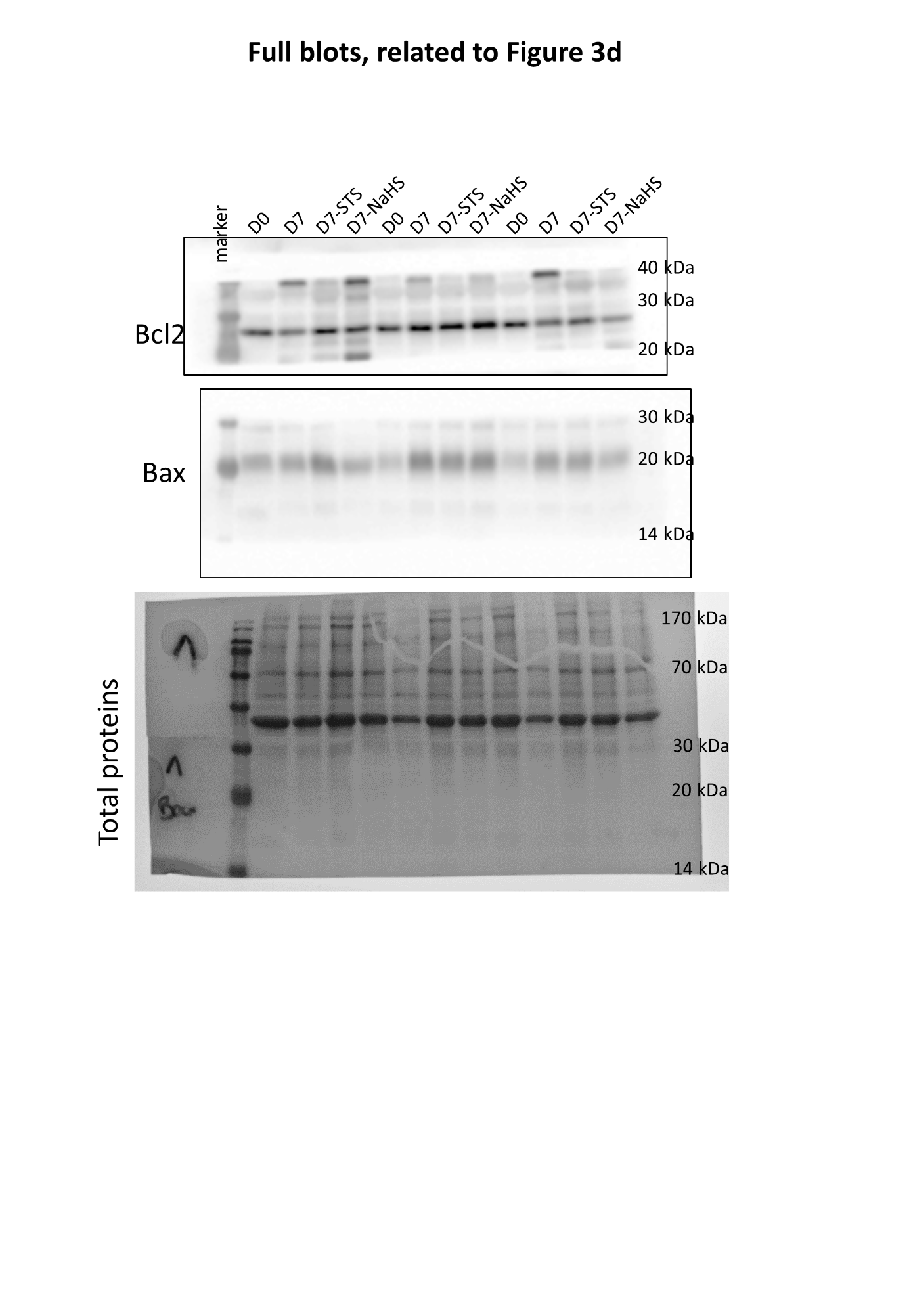

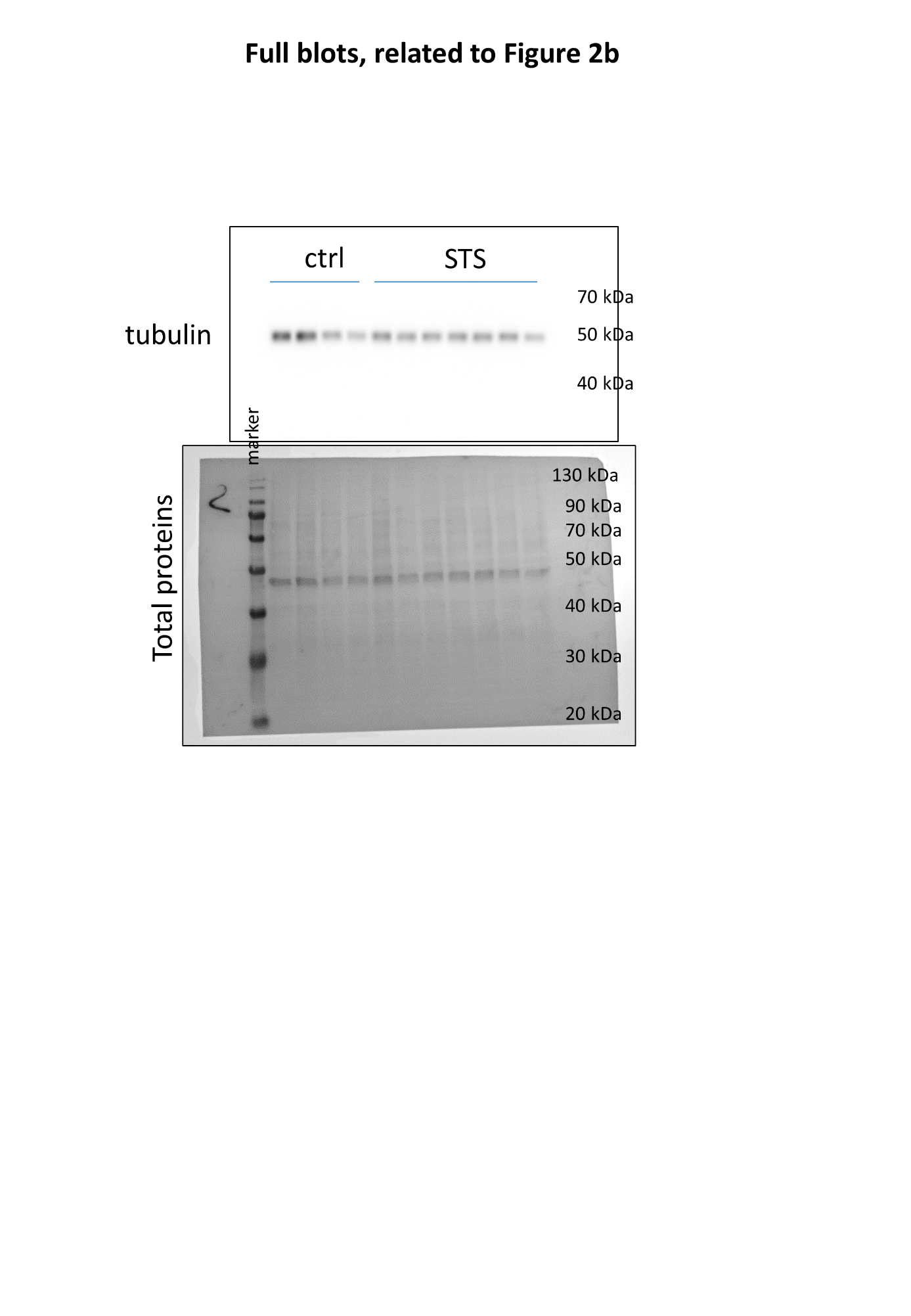

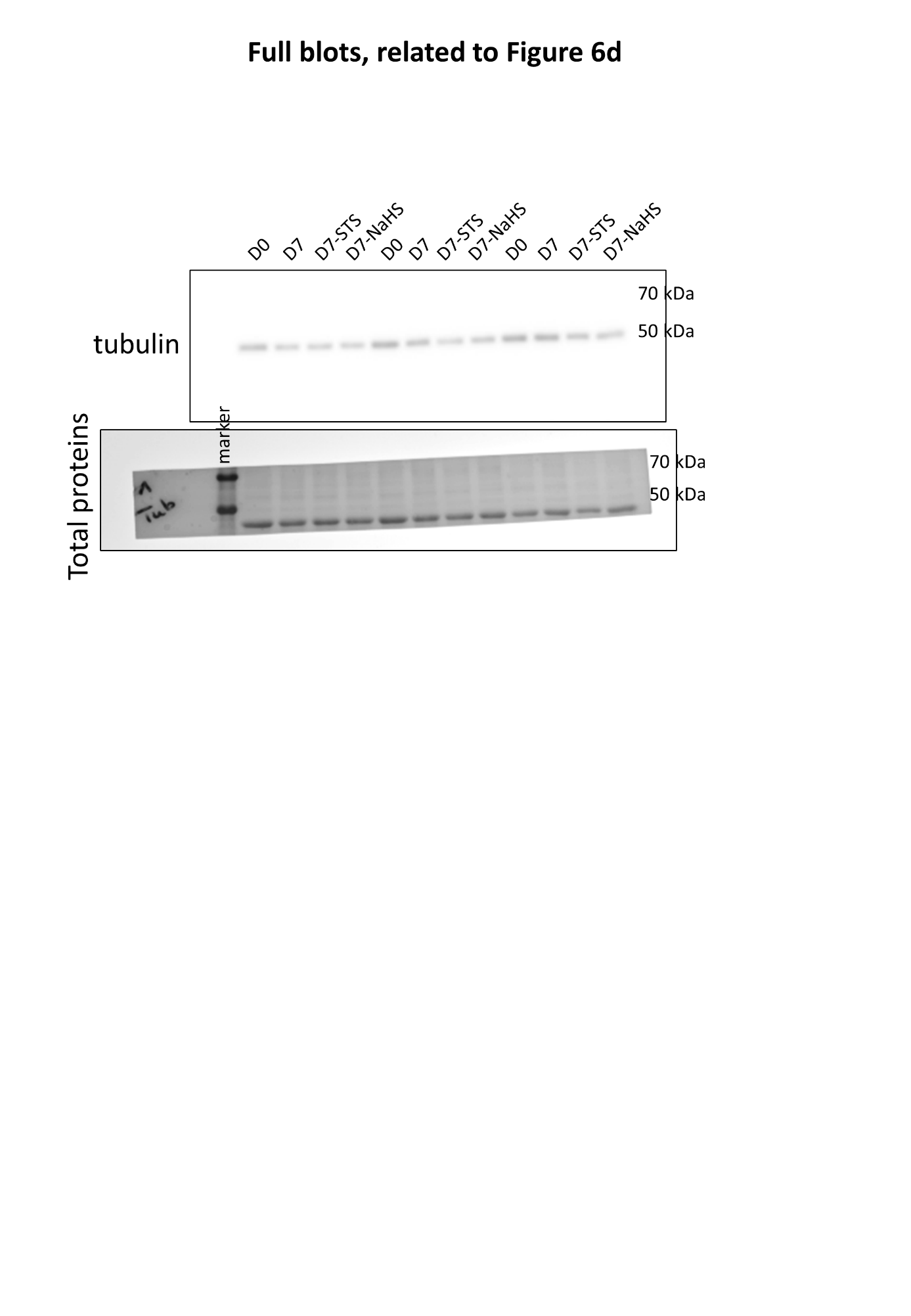

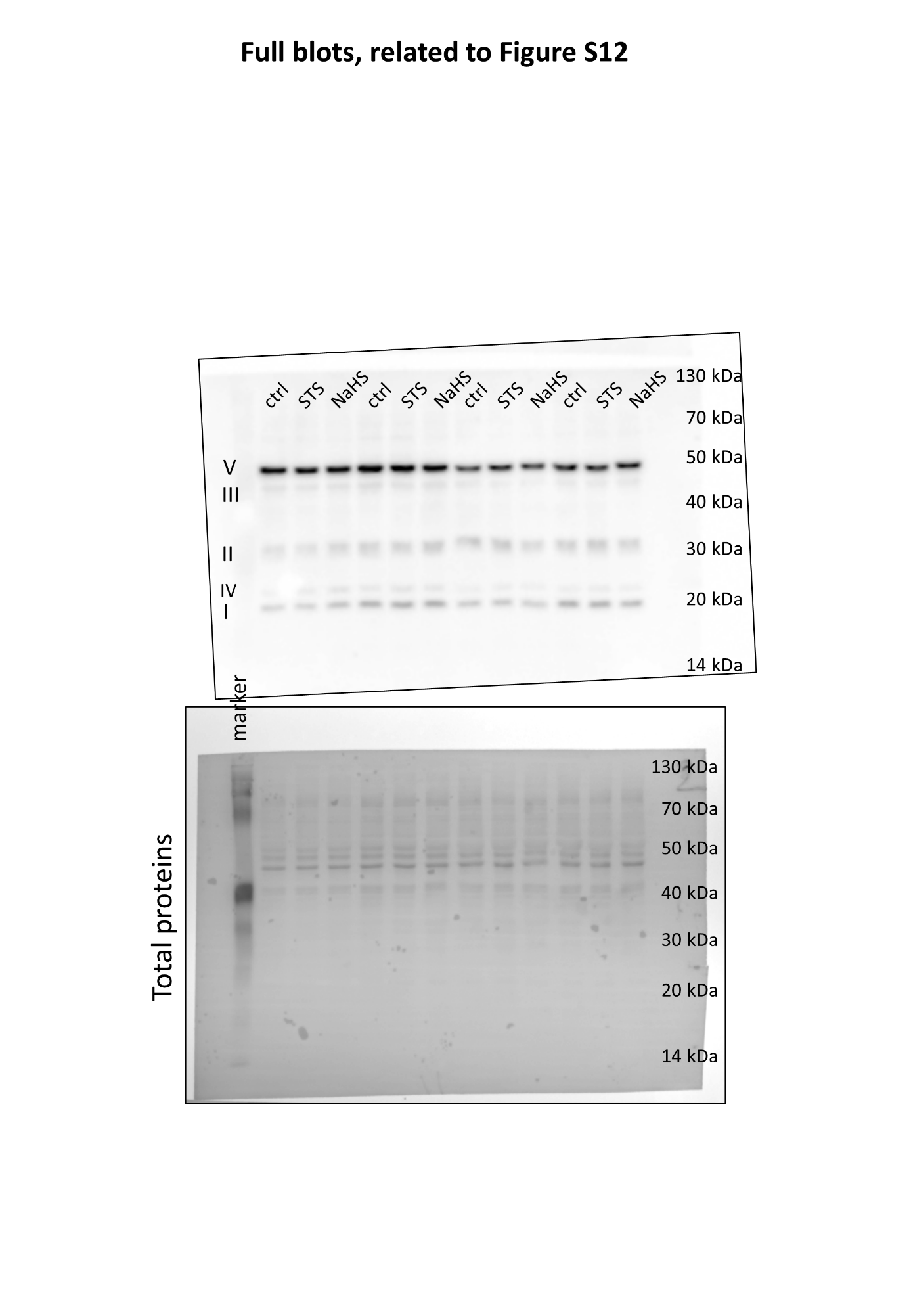

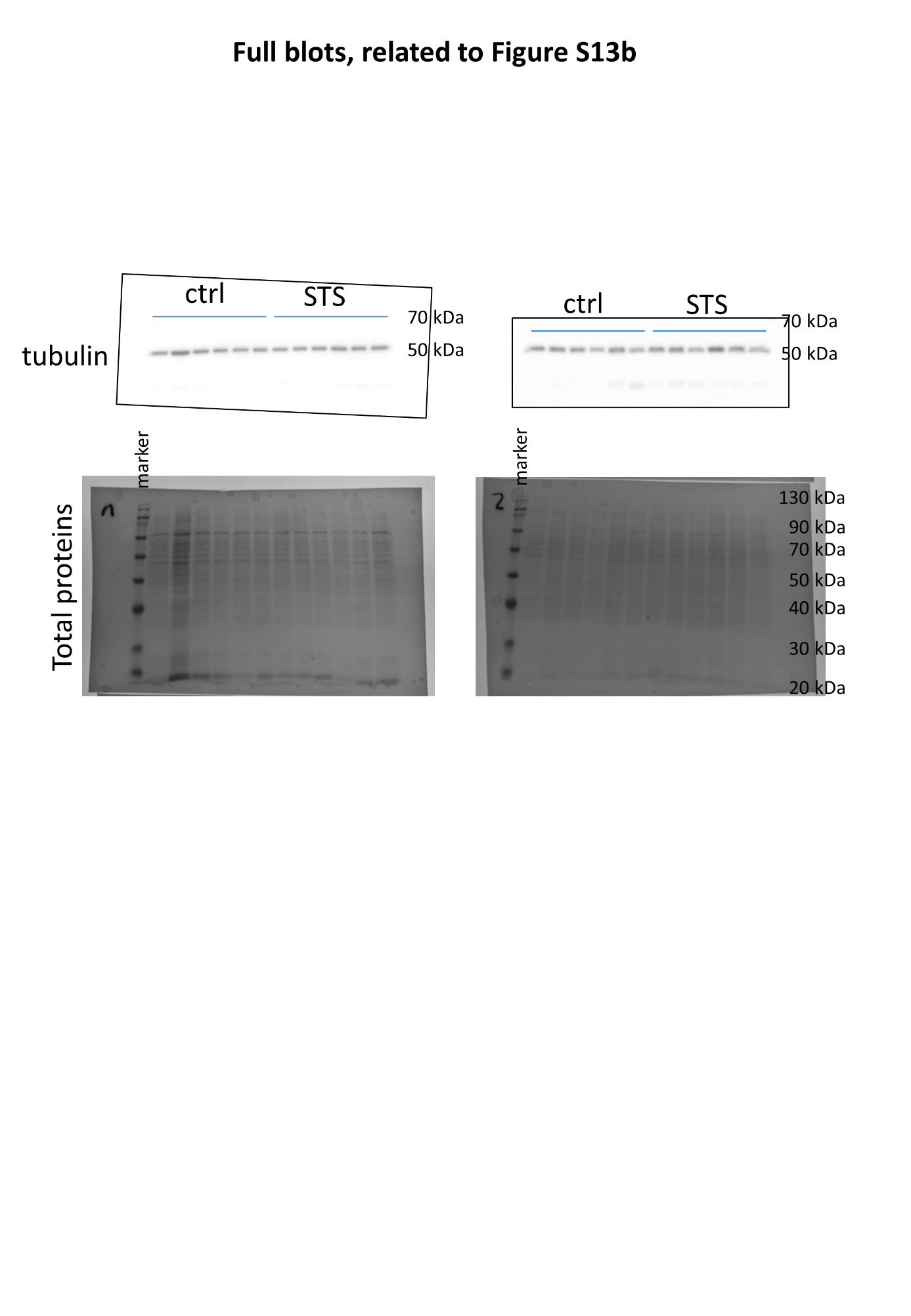

Supplement: Supplementary file 3 [file mmc3.docx]
